# Increased GluK1 Subunit Receptors in Corticostriatal Projection from the Anterior Cingulate Cortex Contributed to Seizure‐Like Activities

**DOI:** 10.1002/advs.202308444

**Published:** 2024-09-03

**Authors:** Xu‐Hui Li, Wantong Shi, Zhi‐Xia Zhao, Takanori Matsuura, Jing‐Shan Lu, Jingmin Che, Qi‐Yu Chen, Zhaoxiang Zhou, Man Xue, Shun Hao, Fang Xu, Guo‐Qiang Bi, Bong‐Kiun Kaang, Graham L. Collingridge, Min Zhuo

**Affiliations:** ^1^ Center for Neuron and Disease Frontier Institute of Science and Technology Xi'an Jiaotong University Xi'an Shaanxi 710049 China; ^2^ Department of Physiology Faculty of Medicine University of Toronto Medical Science Building, 1 King's College Circle Toronto Ontario M5S 1A8 Canada; ^3^ Department of Orthopaedics School of Medicine University of Occupational and Environmental Health Yahatanishi‐ku Kitakyushu 807–8555 Japan; ^4^ Shaanxi Provincial Key Laboratory of Infection and Immune Diseases Shaanxi Provincial People's Hospital Xi'an Shaanxi 710068 China; ^5^ CAS Key Laboratory of Brain Connectome and Manipulation Interdisciplinary Center for Brain Information The Brain Cognition and Brain Disease Institute Shenzhen Institute of Advanced Technology Shenzhen Guangdong 518055 China; ^6^ Department of Neurology First Affiliated Hospital of Guangzhou Medical University Guangzhou Guangdong 510130 China; ^7^ Department of Pharmacology Qingdao University School of Pharmacy Qingdao Shandong 266071 China; ^8^ Department of Biological Sciences College of Natural Sciences Seoul National University Seoul 151–746 South Korea

**Keywords:** corticostriatal projection, anterior cingulate cortex, kainate receptor, seizure, AC1, striatum

## Abstract

The corticostriatal connection plays a crucial role in cognitive, emotional, and motor control. However, the specific roles and synaptic transmissions of corticostriatal connection are less studied, especially the corticostriatal transmission from the anterior cingulate cortex (ACC). Here, a direct glutamatergic excitatory synaptic transmission in the corticostriatal projection from the ACC is found. Kainate receptors (KAR)‐mediated synaptic transmission is increased in this corticostriatal connection both in vitro and in vivo seizure‐like activities. GluK1 containing KARs and downstream calcium‐stimulated adenylyl cyclase subtype 1 (AC1) are involved in the upregulation of KARs following seizure‐like activities. Inhibiting the activities of ACC or its corticostriatal connection significantly attenuated pentylenetetrazole (PTZ)‐induced seizure. Additionally, injection of GluK1 receptor antagonist UBP310 or the AC1 inhibitor NB001 both show antiepileptic effects. The studies provide direct evidence that KARs are involved in seizure activity in the corticostriatal connection and the KAR‐AC1 signaling pathway is a potential novel antiepileptic strategy.

## Graphical abstract

This study found that there is a direct glutamatergic projection from the ACC to the dorsal striatum mediated by AMPA/KA receptors. KAR‐mediated EPSCs increased in the ACC‐striatum pathway following seizure‐like activity in both in vitro and in vivo seizure models. Inhibiting the projection of ACC to the striatum significantly attenuated PTZ‐induced seizure‐like activities. The KAR‐AC1 signaling pathway thus presents a potential antiepileptic target.

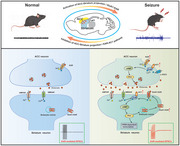

## 1 Introduction

Seizure is an alarming neurological disorder characterized by the occurrence of spontaneous recurrent, which is associated with significant cerebral dysfunction and mortality.^[^ ^1^, ^2^ ^]^ Due to a limited understanding of the underlying molecular and synaptic mechanisms, clinical treatments for epilepsy and seizures remain constrained. Numerous central brain regions, such as the hippocampus, amygdala, striatum, and frontal cortex, have been implicated in seizures.^[^ ^3^, ^4^, ^5^, ^6^ ^]^ Alterations in neuronal excitability and/or the balance of excitation‐inhibition are proposed as key mechanisms of seizure generation.^[^ ^7^ ^]^ For instance, in animal models of seizure, seizure‐like activity can be acutely induced by blocking inhibitory synaptic transmission or by activating excitatory synaptic transmission.^[^ ^8^, ^9^ ^]^ Clinically, antiepileptic drugs effectively control seizures by inhibiting voltage‐gated Na^+^ or Ca^2+^ channels to modify neuronal excitability.^[^ ^10^, ^11^ ^]^ However, due to its poor selectivity, these drugs often cause many central nervous system side effects.

Among various potential antiepileptic drug targets, the kainate receptor (KAR) stands out as a particularly promising candidate.^[^ ^9^, ^12^, ^13^, ^14^ ^]^ In hippocampal mossy fiber synapses, transsynaptic modulation of KAR functions by C1q‐like proteins offers significant potential as an antiepileptic strategy.^[^ ^15^ ^]^ Studies have shown that GluK2/GluK5 receptors contribute to sustained depolarization during repetitive activity.^[^ ^16^ ^]^ Ectopic GluK2/GluK5 receptors are crucial in the physiopathology of the chronic phase of temporal lobe epilepsy.^[^ ^17^ ^]^ Furthermore, antagonists of GluK1containing KARs have been found to prevent pilocarpine‐induced limbic seizures in both in vitro hippocampal slices and in vivo freely moving rats.^[^ ^18^ ^]^ In the amygdala, a specific GluK1 agonist ATPA (2‐Amino‐3‐(5‐(tert‐butyl)‐3‐oxo‐2,3‐dihydroisoxazol‐4‐yl)propanoic acid) can reverse the kindling‐induced impairment of long‐term potentiation (LTP) in the lateral amygdala.^[^ ^19^ ^]^ However, high‐concentration ATPA induces spontaneous epileptiform bursts in rat amygdala slices,^[^ ^20^ ^]^ and GluK1 antagonists prevent hippocampal seizures caused by pilocarpine or electrical stimulation in rats.^[^ ^18^ ^]^ Systemic administration of ATPA in vivo induced seizures in the hippocampus and the amygdala, an effect that was abolished in *GluK1* ^−/−^ mice.^[^ ^21^ ^]^

Anterior cingulate cortex (ACC)^[^ ^8^, ^22^, ^23^ ^]^ and striatum^[^ ^24^, ^25^ ^]^ have been implicated in various forms of seizure. Recent reports have identified KARs in both regions.^[^ ^23^, ^26^, ^27^ ^]^ In the ACC, KARs have been reported to contribute to excitatory synaptic transmission and modulate presynaptic transmitter release.^[^ ^28^ ^]^ Additionally, an NMDAR ‐independent, KAR‐dependent presynaptic form of LTP has been reported.^[^ ^28^, ^29^, ^30^ ^]^ The downstream calcium‐stimulated adenylyl cyclase subtype 1 (AC1) is involved in the roles of KARs in the ACC, which produce cyclic adenosine‐monophosphate (cAMP). Furthermore, recent genetic studies suggest that KARs also play a role in corticostriatal functions.^[^ ^27^, ^31^ ^]^

Previous studies have suggested that seizure‐generating or propagating may involve multiple brain structures and neuronal circuits.^[^ ^32^, ^33^, ^34^, ^35^ ^]^ Recent research has indicated that cortical (somatosensory)‐striatal excitatory connections may also contribute to epilepsy.^[^ ^36^ ^]^ Although the roles of ACC in frontal lobe epilepsy have been recently investigated, the potential involvement of ACC‐striatum projections in seizure has not been explored. In the present study, we combined optogenetic, pharmacological, electrophysiological, genetic, and behavioral approaches to elucidate the role of KARs in corticostriatal connections from the ACC. We found that the role of KARs in corticostriatal projection from the ACC is upregulated following seizure‐like activities. Inhibition of this pathway and the role of KARs attenuated in vivo seizure activities. Our results suggest that KARs and AC1 may be potential therapeutic targets of antiepileptic drugs and neuroprotective agents.

## 2 Results

### 2.1 Glutamatergic Projection from the ACC to the Dorsal Striatum

**Figure 1:**
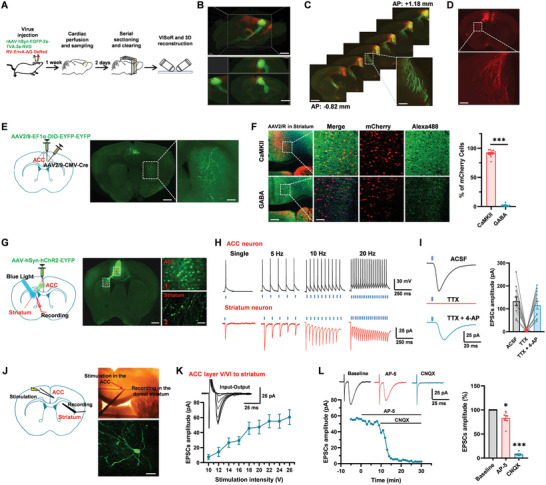
The dorsal striatum neuron received glutamatergic direct descending projection from the ACC. A) The procedure for anterograde and retrograde tracing projections of the ACC using VISoR technology and 3D reconstruction. B) Upper: the lateral view of whole‐brain distribution from ACC inputs and outputs (red: DsRed^+^; green: EGFP^+^). Bottom: the horizontal, sagittal, and coronal views for one 3D‐reconstructed brain from the ACC to the striatum. scale bar, 500 µm. C) Fluorescence images of the brain slice containing ACC and striatum after injection of AAV helper and RV into the ACC, scale bar, 500 and 100 µm. D) The mCherry‐labeled retrograde neurons in the striatum, scale bar, 100 µm. E) Schematic of the viral injection site in the ACC for sparsely labeling experiment (left). Fluorescence images of the brain slice from the ACC‐striatum sparsely labeled (middle and right), scale bar: 200 µm (middle) and 100 µm (right). F) Retrograde adeno‐associated virus AAV2/R‐hSyn‐mCherry injected into the striatum in C57 mice. Immunostaining and statistics showing the colocalization of mCherry‐positive neurons in the ACC were mainly CaMKII‐positive neurons, *n* = 14 – 18 slices/4 mice, ***p < 0.001, unpaired *t*‐test; left scale bar, 100 µm; right scale bar, 20 µm. G) Schematic diagram and representative photomicrograph showing the viral injection site in the ACC, light stimulation site, and whole‐cell patch‐recording placement in the striatum, scale bar: 200 µm (left) and 20 µm (right). H) Blue light stimulation (473 nm) evoked action potentials in the ACC neuron at the I‐clamp configure and evoked EPSCs in the dorsal striatum neuron at the V‐clamp configure at single, 5, 10, and 20 Hz for 1 s. I) Blue light‐induced EPSCs were recorded in striatum neurons in ACSF and after the sequential application of TTX (1 µM) and 4‐AP (100 µM), *n* = 11 neurons/4 mice. J) Schematic diagram and representative recording diagram showing the placement of stimulating electrode in the ACC and recording electrode in the dorsal striatum (left and right upper). The morphological properties of neurons in the striatum were labeled by biocytin during recording (right bottom), scale bar: 20 µm. K) Sample traces and pooled data showed the input‐output relationship of basal EPSCs in the striatum neuron after stimulating the ACC (*n* = 7 neurons/3 mice). L) EPSCs blocked by NMDA receptor antagonist AP‐5 (50 µM) and AMPA/KA receptor antagonist CNQX (20 µM) together within the presence of GABA_A_ receptor antagonist picrotoxin (100 µM) in the ACC to striatum synaptic transmission. Sample time course points (left) and statistical results (right) showed the EPSCs in the presence of CNQX and AP‐5 (*n* = 7 neurons/4 mice, *p < 0.05 and ***p < 0.001, unpaired *t*‐test). error bars indicated SEM.

The striatum, as a basic part of the cortical‐striatal‐thalamic loop circuit, receives glutamatergic excitatory afferents from the entire cortices.^[^ ^37^, ^38^, ^39^ ^]^ In this study, we used the anterograde and retrograde virus to investigate the projection pathway from the anterior cingulate cortex (ACC) to the striatum (**Figure** **1**). We injected AAV‐hSyn‐EGFP‐2a‐TVA‐2a‐RVG as a helper virus into the right ACC in mice. The rabies virus (RV)‐EnvA‐ΔG‐DsRed virus was injected into the same site of ACC after the AAV helper expression for 21 days. After 7 days of RV injection, the mice were perfused and imaged (Figure 1A). By applying the Volumetric Imaging with Synchronized on‐the‐fly‐scan and Readout (VISoR) imaging and 3D reconstruction, we obtained images of the whole brain and different sections of the mouse unilateral ACC inputs and outputs (Figure 1B–D). Abundant direct projection fibers from the ACC were found in the striatum. The virus‐infected varicose fibers and terminals were primarily distributed in the dorsomedial striatum, with few in the dorsolateral and ventral striatum (Figure 1C). The afferent fibers were mainly distributed in the ipsilateral striatum, with only a few on the contralateral side. Additionally, few retrograde neurons were found in the ACC, suggesting that the ACC also received the projecting input from the striatum neurons (Figure 1D). To trace down the single neuron projection at a more precise level, we applied the sparse‐labeling approach. Three weeks after the virus AAV2/9‐EF1α‐DIO‐EYFP‐EYFP and AAV2/9‐CMV‐Cre were injected into the right ACC, more clarity fibers were detected in the ipsilateral striatum (Figure 1E). To test which kind of neuron in the ACC projected to the striatum, we applied retrograde adeno‐associated virus AAV2/R‐hSyn‐mCherry in the striatum. Combining with immunohistochemistry, we found that 91.4% of the ACC neurons projected to the striatum are CaMKII‐positive neurons, suggesting that the ACC‐projecting neurons are excitatory (Figure 1F).

Next, we combined the optogenetic approach and whole‐cell patch‐clamp recording to test the mono‐synaptic transmission between the ACC and striatum (Figure 1G,H). The AAV‐hSyn‐hChR2‐EYFP virus was injected into one side of the ACC. In the ACC neurons, light stimulation can induce action potentials in the current clamp configuration with single, 5 light pulses at 5 Hz, 10 light pulses at 10 Hz, and 20 light pulses at 20 Hz (Figure 1H). We then patched the dorsal striatum neurons and blue light (473 nm) stimulated the virus‐infected fibers in the striatum. Optic‐excitatory postsynaptic currents (EPSCs) were recorded in ipsilateral dorsal striatum neurons after repetitive light stimulation in the striatum (Figure 1H). The EPSCs were abolished after bath application of tetrodotoxin (TTX) (1 µM) but then partially rescued by a 4‐aminopyridine (4‐AP) (100 µM) bath application, further confirming the monosynaptic connection between the ACC and striatum (Figure 1I).

Due to the ChR2 failing to follow 200 Hz high‐frequency stimulation and the expression of the optogenetic virus not stable in each neuron, the classical electrical stimulations were applied with a bipolar tungsten electrode placed in the different layers of the ACC to explore characteristics of synaptic transmission in the ACC to the striatum (Figure 1J). We found that stimulating both layer II/III and layer V/VI in the ACC can evoke reliable EPSCs in the dorsal striatum neurons. Then, we recorded the input (stimulation intensity)‐output (EPSCs amplitude) (I‐O curves) relationship of the striatum neurons by stimulating the layer V/VI of the ACC (Figure 1K). We found the amplitudes of these EPSCs increased in a stimulation intensity‐dependent manner. To test whether the excitatory synaptic transmission is mediated by glutamate receptors, the AMPA/KA receptor antagonist 6‐cyano‐7‐nitroquinoxaline‐2, 3‐dione (CNQX, 20 µM) and NMDA receptor antagonist D‐2‐amino‐5‐phosphonopentanoic acid (AP‐5, 50 µM) were bath applied. The EPSCs were rapidly and largely blocked by following application of AP‐5 and CNQX (AP5: 83.1% ± 5.2% of baseline; CNQX: 6.8% ± 2.1% of baseline; *n* = 7 neurons/4 mice; Figure 1L). Taken together, these results indicate that there is a direct glutamatergic excitatory synaptic transmission from the ACC to the striatum. These synaptic transmissions are primarily mediated by the AMPA/KA receptor, with less mediated by the NMDA receptor.

### 2.2 Kainate Receptor‐Mediated Synaptic Transmission in Corticostriatal Projection from the ACC

**Figure 2:**
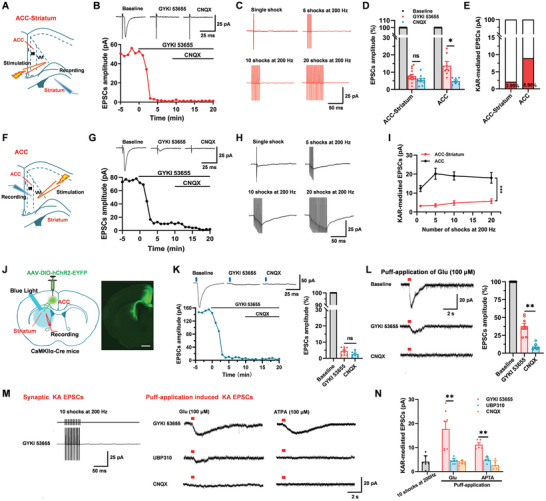
KAR‐mediated EPSCs in the ACC‐dorsal striatum pathway. A and F) Schematic diagram showing the placement of stimulating electrode in layer V/VI of the ACC and recording electrode in the dorsal striatum A) and interior ACC F). B and G) In the presence of picrotoxin (100 µM) and AP‐5 (50 µM), KAR‐mediated EPSCs were observed after the application of a potent AMPA receptor antagonist GYKI 53655 (100 µM) and then blocked by AMPA/KA receptor antagonist CNQX (20 µM) in the ACC‐striatum (B) and interior ACC (G). Sample traces (top) and sample time course points (bottom) showed the EPSCs in the presence of GYKI 53655 and CNQX. C and H) Representative traces of KA receptor‐mediated EPSCs were obtained after the application of different numbers of stimulation (1, 5, 10, and 20 shocks) at 200 Hz in the presence of GYKI 53655. D) Summary results showed CNQX blocked KAR‐mediated EPSCs in the interior ACC but not in the ACC‐striatum (ACC‐striatum: *n* = 14 neurons/5 mice, *p *> 0.05, paired *t*‐test; ACC: *n* = 7 neurons/3 mice; *p < 0.05, paired *t*‐test). E) The percentage of KAR‐mediated EPSCs in basal synaptic transmission in the ACC‐striatum and interior ACC synapses. I) Statistical results showed that the peak amplitude of the KAR‐mediated EPSCs in the ACC‐striatum was smaller than those in the interior ACC by repetitive stimulations at 200 Hz (striatum: *n* = 13 neurons/5 mice; ACC: *n* = 10 neurons/4 mice; *F*_(1, 92)_ = 132.22, ***p < 0.001, two‐way ANOVA). J) Schematic diagram and representative photomicrograph showing the viral injection site in the ACC, light stimulation site, and whole‐cell patch‐recording placement in the striatum in CaMKIIα‐Cre mice, scale bar: 200 µm. K) Optical‐induced KAR‐mediated EPSCs in the ACC to striatum synapses in the presence of GYKI 53655 and CNQX (*n* = 9 neurons/3 mice, *p *> 0.05, paired *t*‐test). L) Sample traces (left) and statistical results (right) show that post‐synaptic currents evoked by puff‐application of glutamate (100 µM) were dramatically decreased by GYKI 53655 in the striatum neuron in the presence of picrotoxin and AP‐5, but still had residual currents. These residual currents can be completely blocked by CNQX (*n* = 6–8 neurons/4 mice, **p < 0.01, unpaired *t*‐test). M) Sample traces showed that no synaptic KAR‐mediated EPSCs in the striatum neuron were evoked by 10 shocks (200 Hz) of electrical stimulation in the presence of GYKI 53655 (top). Puff‐application of glutamate (100 µM) and GluK1 agonist ATPA (100 µM) both can induce KAR‐mediated currents. These KAR‐mediated currents were blocked by a specific GluK1 antagonist UBP310 (10 µM) and CNQX (bottom). N) Statistical results showing the amplitude of KAR‐mediated EPSCs evoked by 10 shocks (200 Hz) electrical stimulation, puff‐application of glutamate and ATPA in the presence of GYKI 53655, UBP310 and CNQX (glutamate: *n* = 7 neurons/3 mice, **p < 0.01, paired *t*‐test; ATPA: *n* = 5 neurons/3 mice, **p < 0.01, paired *t*‐test). ns. means no significant difference, error bars indicated SEM.

Kainate receptor (KAR) is one of the ion glutamate receptors, that have been found to play roles in synaptic transmission in the central synapses, such as the ACC, striatum, amygdala, and hippocampus.^[^ ^28^, ^31^, ^40^, ^41^, ^42^, ^43^ ^]^ We wonder whether KARs contribute to synaptic responses in corticostriatal connections (**Figure** **2**A). EPSCs were recorded in the presence of picrotoxin (100 µM) and AP‐5 (50 µM) with a single‐pulse stimulation in the ACC. A potent AMPA receptor antagonist GYKI 53655 (100 µM) was bath applied to isolate KAR‐mediated EPSCs after recording steady basal EPSCs for 5 min. As shown in Figure 2B, GYKI 53655 rapidly and almost completely reduced the amplitude of EPSCs in the dorsal striatum neuron by stimulation in the ACC. There was no sustainable decrease after applying CNQX (Baseline: −42.9 ± 6.3 pA; GYKI 53655: −3.1 ± 0.4 pA, CNQX: ‐2.1 ± 0.3 pA; Figure 2B,D). However, in the interior ACC, there were remarkable KAR‐mediated EPSCs in the ACC neurons after applying GYKI 53655 which can be blocked by CNQX (Baseline: −89.4 ± 2.3 pA; GYKI 53655: −12.3 ± 1.4 pA, CNQX: −4.2 ± 0.6 pA; Figure 2G,D). The KAR‐mediated EPSCs are 2.09% of basal synaptic transmission in the ACC to striatum synapses, and 8.96% of basal synaptic transmission in the ACC synapses (Figure 2E).

As previous studies showed in most synapses, brief repetitive impulse trains increased KAR‐mediated EPSCs.^[^ ^43^, ^44^ ^]^ To determine the summarized amplitude of KAR‐mediated EPSCs, repetitive stimulations were applied for single, 5, 10, and 20 shocks at 200 Hz in the presence of GYKI 53655 in the striatum neurons by stimulating the ACC. As shown in Figure 2C, the amplitudes of KAR‐mediated EPSCs were not significantly increased with repetitive stimuli in striatum neurons but dramatically increased in the ACC neurons (Figure 2C,H,I). These results suggest smaller KAR‐mediated EPSCs in the ACC‐striatum synapses compared to those in the interior ACC. High‐frequency repetitive stimulation also failed to enhance KAR‐mediated EPSCs in the ACC‐striatum synapses.

To further confirm the KAR‐mediated synaptic transmission in ACC‐striatum synapses, we also combined the AAV‐DIO‐hChR2‐EYFP virus and CaMKIIα‐Cre mice to selectively stimulate the ACC‐striatum glutamatergic projection (Figure 2J). Optical‐induced EPSCs were rapidly and completely reduced by GYKI 53655 and not significantly changed after applying CNQX (Figure 2K). These results are consistent with the data from electric‐induced EPSCs. Next, to further confirm the roles of KARs in ACC‐striatum synapses, we recorded puff‐application of glutamate‐induced post‐synaptic currents in striatum neurons. We found that puff‐application of glutamate‐induced post‐synaptic currents which were largely reduced by GYKI 53655, but still have a small residual current that can be blocked by AMPA receptor/KAR antagonist CNQX (Baseline: −72.1 ± 6.3 pA; GYKI 53655: −23.8 ± 5.2 pA, 33.0% ± 7.2% of baseline; CNQX: −4.4 ± 0.6 pA, 6.1% ± 0.9% of baseline; Figure 2L). These results indicate that puff‐application of glutamate‐induced KAR‐mediated currents in the striatum neurons. Moreover, these KAR‐mediated currents can be induced by puff‐application of glutamate even if there were no synaptic KAR‐mediated currents by repetitive electrical stimulation of the ACC (10 shocks at 200 Hz, Figure 2M). These currents were blocked by specific GluK1 antagonist UBP310 (10 µM) and CNQX (Figure 2M,N). In addition, puff‐application of GluK1 agonist ATPA can also induce KAR‐mediated currents, even without synaptic KAR‐mediated EPSCs, and blocked completely by UBP310 and CNQX (Figure 2M,N). In summary, these results suggest that there is an extra‐synaptic GluK1‐containing KA receptor in striatum neurons.

### 2.3 In vitro, Seizure‐Like Activity Increased KAR‐Mediated EPSCs in the ACC‐Striatum

**Figure 3:**
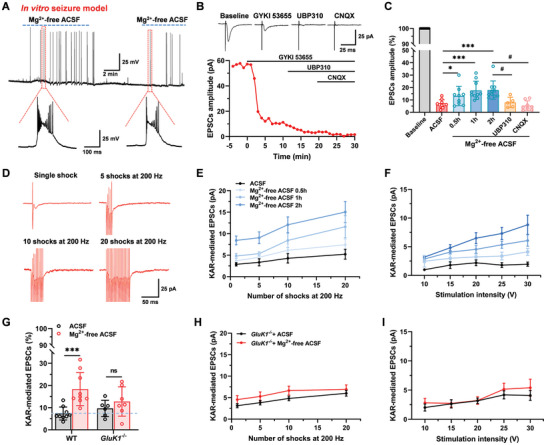
KAR‐mediated EPSCs increased after Mg^2+^‐free ACSF‐induced in vitro seizure‐like activity. A)The sample traces showing seizure‐like activity were induced by perfusion of Mg^2+^‐free ACSF in the striatum neuron at the I‐clamp. B) Sample traces (upper) and sample time course points (bottom) show the EPSCs in the presence of GYKI 53655, UBP310, and CNQX in the striatum neuron by stimulating the ACC after 2 h seizure‐like activity. C) Statistical results show the percentage of KAR‐mediated EPSCs amplitude after the perfusion of Mg^2+^‐free ACSF for 0.5, 1, 2, and 2 h with in presence of UBP310 and CNQX in the striatum neuron by stimulating the ACC. Perfusion with Mg^2+^‐free ACSF was time‐dependently increased KAR‐mediated EPSCs and blocked by UBP310 and CNQX (ACSF: *n* = 10 neurons/4 mice; Mg^2+^‐free ACSF for 0.5 h, *n* = 9 neurons/4 mice; 1 h, *n* = 10 neurons/4 mice; 2 h, *n* = 10 neurons/4 mice; 2 h + UBP310, *n* = 5 neurons/3 mice; 2 h + CNQX, *n* = 8 neurons/3 mice; *p < 0.05 and ***p < 0.001, * means compared with ACSF; ^#^ *p *< 0.05, ^#^ means compared with Mg^2+^‐free ACSF 2 h). D) Representative traces of KAR‐mediated EPSCs were obtained after the application of different numbers of stimuli (1, 5, 10, and 20 shocks) at 200 Hz after 2 h of seizure‐like activity. E) Summary results showing the peak amplitude of the KAR‐mediated EPSCs by repetitive stimulations (200 Hz) after perfusing Mg^2+^‐free ACSF for 0.5, 1, and 2 h in the ACC‐striatum synapses, *n* = 6–7 neurons/3‐4 mice. F) Pooled data showing the input‐output relationship of KAR‐mediated EPSCs after perfusing Mg^2+^‐free ACSF for 0.5, 1, and 2 h. G) Statistical results show the percentage of KAR‐mediated EPSCs in wild‐type (WT) mice and *GluK1* ^−/‐^ mice. KAR‐mediated EPSCs were no increased after perfusing Mg^2+^‐free ACSF for 2 h in the *GluK1* ^−/−^ mice (WT: *n* = 9 neurons/3 mice, ***p < 0.001, unpaired *t*‐test; *GluK1* ^−/−^: *n* = 6–7 neurons/3 mice, *p* > 0.05, unpaired *t*‐test). H) Statistical results show the amplitude of the KAR‐mediated EPSCs by repetitive stimulations (200 Hz) after perfusing Mg^2+^‐free ACSF for 2 h in the *GluK1* ^−/−^ mice (*n* = 8–13 neurons/3‐4 mice, *F*_(1, 76)_ = 0.49, *p *> 0.05, two‐way ANOVA). I) Statistical results show the input‐output relationship after perfusing Mg^2+^‐free ACSF for 2 h in the *GluK1* ^−/−^ mice (*F*_(1, 95)_ = 0.34, *p *> 0.05, two‐way ANOVA). Error bars indicated SEM.

Previous studies have shown that KARs have an important role in epilepsy. KARs agonist kainic acid (KA) is widely used to induce behavioral seizures.^[^ ^33^, ^42^ ^]^ Thus, we wonder whether the role of KARs was changed in the ACC‐striatum pathway after seizure‐like activities. The Mg^2+^‐free artificial cerebrospinal fluid (ACSF) was employed to induce seizure‐like activity in the brain slices as previous studies reported,^[^ ^45^, ^46^ ^]^ (**Figure** **3**). As shown in Figure 3A, seizure‐like activities in dorsal striatum neurons were induced by Mg^2+^‐free ACSF after perfusing for ≈15 min. These seizure‐like activities gradually disappeared when washed with normal ACSF, but appeared after perfusing with Mg^2+^‐free ACSF again. We found that KAR‐mediated EPSCs were significantly increased after perfusing Mg^2+^‐free ACSF with different perfusion times (0.5, 1, and 2 h) compared with a normal ACSF group in the ACC‐striatum synapses. KARs contributed 13.1% ± 2.7% (−4.1 ± 0.7 pA), 17.7% ± 2.4% (−5.8 ± 1.0 pA), and 18.8% ± 2.3% (−8.2 ± 1.0 pA) of the AMPA/KAR‐mediated EPSCs after 0.5 h, 1 h, and 2 h perfusing Mg^2+^‐free ACSF compared with normal ACSF 6.1 ± 1.4% (‐2.6 ± 0.5 pA), respectively. Application of specific GluK1 antagonist UBP310 (10 µM) and CNQX blocked the KAR‐mediated EPSCs induced by seizure‐like activities (Figure 3B,C). The KAR‐mediated EPSCs were able to show the summary effect after repetitive stimuli with single, 5, 10, and 20 shocks at 200 Hz by stimulating the ACC in Mg^2+^‐free ACSF (Figure 3D,E). In addition, the input‐output curves of KAR‐mediated EPSCs were shifted to the left after perfusing Mg^2+^‐free ACSF for 0.5, 1, and 2 h, compared with the normal ACSF group (Figure 3F).

Next, we assessed the facilitation effect of KAR‐mediated EPSCs in vitro seizure‐like activity in *GluK1* ^−/‐^ mice. We found that *GluK1* ^−/−^ mice failed to exhibit the significant enhancement of KAR‐mediated EPSCs after perfusing with Mg^2+^‐free ACSF for 2 h (ACSF: 9.6% ± 1.5% of the AMPA/KAR‐mediated EPSCs; Mg^2+^‐free ACSF: 13.4% ± 4.5% of the AMPA/KAR‐mediated EPSCs; Figure 3G). Repetitive stimulations had no effect on KAR‐mediated EPSCs and the input‐output curves of KAR‐mediated EPSCs were not changed in *GluK1* ^−/−^ mice compared with in the ACSF group after seizure‐like activities (Figure 3H,I). Taken together, these results suggest that seizure‐like activities enhance KAR‐mediated synaptic transmission in the ACC‐striatum pathway.

### 2.4 The Role of KARs Upregulated in Pentylenetetrazole (PTZ)‐Induced In Vivo Seizure Behavior

**Figure 4:**
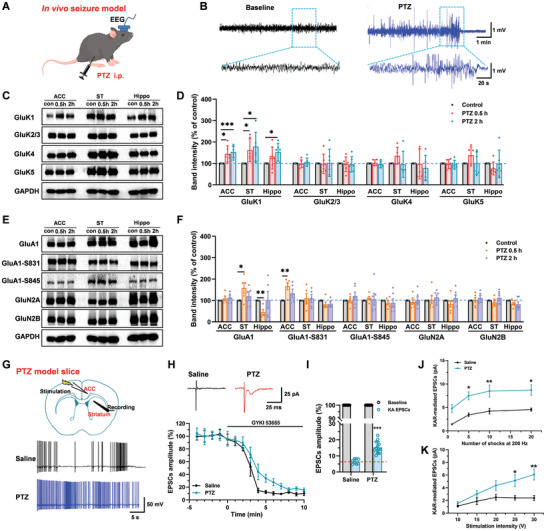
The roles of GluK1 upregulated in the ACC‐striatum pathway after PTZ‐induced in vivo seizure activity. A, B) Schematic diagram and sample trance of EEG recording after intraperitoneal (i.p.) injection of PTZ (50 mg kg^−1^) induced seizure‐like activity in mice. C, D) Representative and statistics western blots of KAR subunits GluK1, GluK2/3, GluK4, and GluK5 expression in total homogenates of the ACC, striatum, and hippocampus, *n* = 5–6. E, F) Representative and statistics western blots of AMPAR subunits GluA1, GluA1‐Ser 831, GluA1‐Ser 845, and NMDAR subunits GluN2A, GluN2B expression in total homogenates of the ACC, striatum, and hippocampus, *n* = 5–6. G) Upper: schematic diagram showing the placement of stimulating electrode in the ACC and recording electrode in the dorsal striatum in PTZ model slice. Bottom: representative trace of action potential firing rate in striatum neuron in saline and PTZ groups. H) The sample traces and summary time course points show the amplitude percentage of EPSCs in the PTZ‐induced seizure and saline mice in the ACC‐striatum (Saline: n = 6 neurons/3 mice; PTZ: *n* = 9 neurons/5 mice). I) Statistical results show that the percentage of KAR‐mediated EPSCs increased in the PTZ group (Saline: *n* = 8 neurons/3 mice; PTZ: *n* = 13 neurons/5 mice; ***p < 0.001, paired *t*‐test). J) Statistical results showing the amplitude of the KAR‐mediated EPSCs evoked by repetitive stimulations (200 Hz) were significantly increased in the PTZ group compared with the saline group (Saline: *n* = 8 neurons/3 mice; PTZ: *n* = 12 neurons/4 mice; *F*_(1, 72)_ = 28.66, *P* < 0.001, two‐way ANOVA). K) Statistical results showing the input‐output curves of the KAR‐mediated EPSCs were shifted to the left in the PTZ group compared with the saline group (Saline: *n* = 8 neurons/3 mice; PTZ: *n* = 9 neurons/4 mice; *F*_(1,75)_ = 11.37, *p *< 0.01, two‐way ANOVA). *p < 0.05, **p < 0.01, and ***p < 0.001, error bars indicated SEM.

After in vitro seizure‐like activities in the brain slices, we want to know whether the roles of KARs were changed in the in vivo seizure model. Pentylenetetrazole (PTZ), a GABA_A_ receptor antagonist, was used to induce an in vivo seizure model, as previously reported.^[^ ^47^, ^48^ ^]^ As shown in **Figure** **4**A,B, PTZ significantly induced the change in electroencephalographic (EEG) in mice. Next, we detected the expression of KAR subunits by western blot approach in the ACC, striatum, and hippocampus of mice after PTZ‐induced in vivo seizure (S1, Supporting Information). The expression of GluK1 subunit was significantly increased in the ACC, striatum, and hippocampus over time after the PTZ injection, but the expression of GluK2/3, GluK4, and GluK5 did not change after PTZ‐injection 2 h in these 3 regions (Figure 4C,D). In addition, we also tested the expression of AMPARs and NMDARs subunits after in vivo seizure. We found that the GluA1 upregulated in the striatum and downregulated in the hippocampus after the PTZ injection at 0.5 h, and phosphatized GluA1(GluA1‐Serine 831) in the ACC upregulated after 0.5 h of the PTZ model. The expression of GluN2A and GluN2B was not changed after the PTZ injection (Figure 4E,F).

Next, we recorded KAR‐mediated synaptic transmission in brain slices after PTZ‐induced seizure. As shown in Figure 4G, the firing rate of the striatum neuron was increased after PTZ‐induced seizure. KAR‐mediated EPSCs in ACC to striatum synapse were significantly increased, and KA receptors contributed 16.1% ± 1.3% of the AMPA/KAR‐mediated EPSCs in the PTZ group compared with 6.6% ± 0.4% in the saline group (Figure 4H,I). Moreover, consistent with the results of in vitro seizure‐like activities, repetitive stimulation also significantly increased KAR‐mediated EPSCs in PTZ‐induced seizure mice (Figure 4J). The input‐output curves of KAR‐mediated EPSCs were significantly shifted to the left in the PTZ‐induced seizure mice (Figure 4K). Taken together, these results suggest that the roles of KARs upregulate both in vitro and in vivo seizure‐like activities.

### 2.5 Inhibiting the Projection from the ACC to the Striatum Attenuated Seizure Behavior

**Figure 5:**
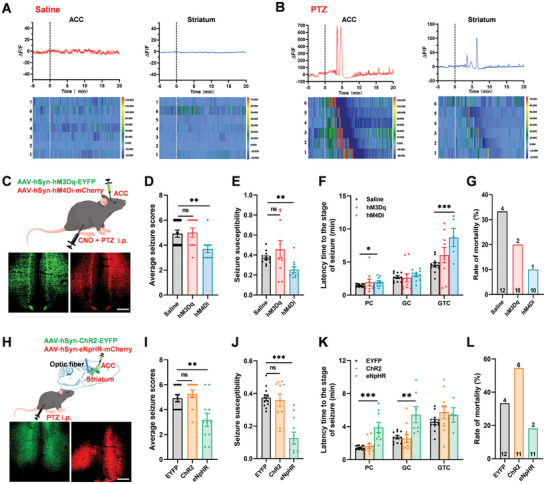
Inhibition of the corticostriatal projection from the ACC attenuated seizure behavior. A) The ΔF/F of representative trace (upper) and heatmaps (bottom) show that Ca^2+^ signals are recorded by fiber‐photometry of the ACC (left) and striatum (right) in mice injected with saline (*n* = 7 mice). B) The ΔF/F of representative trace (upper) and heatmaps (bottom) show that Ca^2+^ signals of the ACC (left) and striatum (right) in mice injected with PTZ (*n* = 6 mice). The colored bar on the right of heatmaps indicates ΔF/F (%). C) Schematic diagram and representative photomicrograph showing the chemical genetics viral injection site in the ACC, and intraperitoneal injection of CNO (1 mg kg^−1^), scale bar: 100 µm. D) Chemical inhibition of the activity of the ACC reduced PTZ‐induced seizure severity scores (Saline, *n* = 12 mice; hM3Dq, *n* = 10 mice, *p* = 0.8576, unpaired *t*‐test; hM4Di, *n* = 10 mice, **p < 0.01, unpaired *t*‐test). E) Chemical inhibition of the activity of the ACC reduced the PTZ‐induced seizure susceptibilities (Saline vs hM3Dq, *p *= 0.3111, unpaired *t*‐test; Saline vs hM4Di, **p < 0.01, unpaired *t*‐test). F) Chemical inhibition of the activity of the ACC increased the latency time to the partial clonus (PC) (Saline vs hM4Di, *p < 0.05, unpaired *t*‐test) and generalized tonic‐clonic (GTC) (Saline vs hM4Di, ***p < 0.001, unpaired *t*‐test) stage of PTZ‐induced seizures. (G) The rate of mortality of mice injected with saline or CNO after PTZ injection. H) Schematic diagram and representative photomicrograph showing the viral injection site in the ACC, and light stimulation site in the striatum, scale bar: 100 µm. I) Yellow light (593 nm) inhibition of the corticostriatal projection from the ACC reduced the PTZ‐induced seizure severity scores (EYFP, *n* = 12 mice; ChR2, *n* = 11 mice, *p *= 0.4044, unpaired *t*‐test; eNpHR, *n* = 11 mice, **p < 0.01, unpaired *t*‐test). J) Seizure susceptibilities were reduced by light inhibition of the corticostriatal projection (EYFP vs ChR2, *p *= 0.7134, unpaired *t*‐test; EYFP vs eNpHR, ***p < 0.001, unpaired *t*‐test). K) Light inhibition of the corticostriatal projection from the ACC increased the latency time to the PC (EYFP vs eNpHR, ***p < 0.001, unpaired *t*‐test) and generalized clonus (GC) stages (EYFP vs eNpHR, **p < 0.01, unpaired *t*‐test). L) The rate of mortality of mice in EYFP, ChR2, and eNpHR groups after blue or yellow light stimulation. n.s means no significant difference, error bars indicated SEM.

Previous studies showed that both ACC and striatum had been indicated in seizures, we then tested whether the ACC‐striatum pathway contributed to the seizure behaviors.^[^ ^22^, ^23^, ^24^ ^]^ First, we recorded the calcium signals in the ACC and striatum by using fiber‐photometry to test whether the ACC and striatum are activated after seizure behavior. AAV2/9‐hSyn‐GCaMP6s‐WPRE‐pA virus was injected into the unilateral ACC, and the optical fibers were implanted into the ipsilateral ACC and striatum. We found that tremendous Ca^2+^ spikes were observed in both ACC and striatum after PTZ injection compared with saline injection (**Figure** **5**A,B). Two Ca^2+^ spikes were detected ≈ 5 min after the PTZ injection in the ACC and three spikes appeared in the striatum. These data suggest that the ACC and striatum may play different roles in the activity of seizure.

Next, we wonder whether activating or inhibiting the activity of ACC affects the seizure behavior by using chemogenetic tools (Figure 5C). In mice, intraperitoneal (i.p.) injection of PTZ (50 mg kg^−1^) can successfully induce behavioral seizures after injection 2–5 min. Clozapine N‐oxide (CNO) intraperitoneal injection combined with hM4Di alleviated the seizure behavior significantly by decreasing the seizure scores and susceptibility (Figure 5D,E), meanwhile increasing the latency time to the stage of seizure and decreasing the rate of mortality (Figure 5F,G). However, the CNO injection combined with hM3Dq did not affect the seizure behavior significantly, which may be due to the ceiling effect that has been reached by PTZ‐induced seizure and activated the ACC cannot enhance the seizure behavior.

To further confirm the role of the ACC‐striatum pathway in seizure behavior, optogenetic modulation was also been carried out to specifical activate or inhibit the ACC‐striatum pathway in the seizure behavior (Figure 5H). By implanting optical fibers in the dorsal striatum, we found that yellow light (593 nm) inhibited the ACC‐striatum pathway and alleviated the seizure behavior significantly by decreasing the seizure scores and susceptibility (Figure 5I–L). Similar to chemogenetic, activation of the ACC‐striatum pathway by blue light was not shown to significantly increase seizure behaviors, although partly aggravated the seizure behaviors. These results indicate that inhibiting the corticostriatal projection from the ACC attenuates seizure behaviors.

### 2.6 Decrease the Roles of GluK1 Shows an Antiepileptic Effect

**Figure 6:**
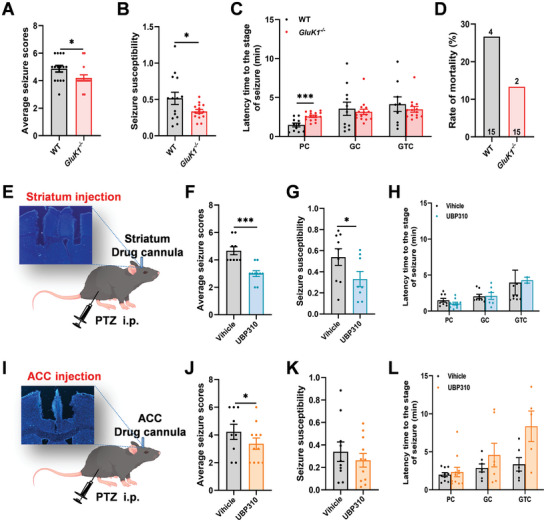
PTZ‐induced seizure behaviors were attenuated in *GluK1* ^−/−^ mice or in mice with an injection of GluK1 antagonist UBP310. A) The PTZ‐induced seizure severity scores were reduced in the *GluK1^−/−^* mice (WT and *GluK1^−/−^*, each *n* = 15 mice, *p < 0.05, unpaired *t*‐test). B) The PTZ‐induced seizure susceptibilities were decreased in the *GluK1^−/−^* mice (*p< 0.05, unpaired *t*‐test). C) The latency time to the PC stage was delayed in *GluK1^−/−^* mice after PTZ‐induced seizure (***p < 0.001, unpaired *t*‐test). D) The rate of mortality induced by PTZ in the *GluK1^−/−^* mice. E) Schematic diagram showing the drug cannula embedded site in the striatum, and intraperitoneal injection of PTZ. F) UBP310 injected into the striatum reduced the PTZ‐induced seizure severity scores (Vehicle, *n* = 9 mice; UBP310, *n* = 10 mice; ***p < 0.001, unpaired *t*‐test). G) UBP310 injected into the striatum reduced the PTZ‐induced seizure susceptibilities (*p < 0.05, unpaired *t*‐test). H) The latency time to the stage of seizures was not changed in mice with striatum‐injected UBP310. I) Schematic diagram showing the drug cannula embedded site in the ACC, and intraperitoneal injection of PTZ. J) UBP310 injected into the ACC reduced the PTZ‐induced seizure severity scores (Vehicle, *n* = 9 mice; UBP310, *n* = 11 mice; *p < 0.05, unpaired *t*‐test). K) UBP310 injected into the ACC did not affect the PTZ‐induced seizure susceptibilities. L) The latency time to the stage of seizures was not changed in mice with ACC‐injected UBP310. Error bars indicated SEM.

Next, we wondered whether inhibition of the functions of KARs in the ACC‐striatum pathway reduces behavioral seizures in vivo. To test this notion, we first investigated PTZ‐induced seizures in the *GluK1* ^−/−^ mice. The PTZ‐induced seizure scores and susceptibility were significantly reduced in the *GluK1^−/−^* mice (**Figure** **6**A,B). The latency time to the PC stage of seizure was increased and the rate of mortality was decreased in *GluK1^−/−^* mice after PTZ‐induced seizures (Figure 6C,D). Considering the possible genetic compensatory changes in genetic deletion mice, we next tested the antiepileptic effect by application of the selective pharmacological GluK1 inhibitor UBP310 in the striatum and the ACC. We found that striatum injection of UBP310 (10 µM) can significantly decrease the seizure score and susceptibility (Figure 6F,G). In the ACC, injection of UBP310 can also decrease the seizure score (Figure 6J,K). It is worth noting that injection of UBP310 does not affect the latency time to the stage of seizure both in the ACC and striatum (Figure 6H,L). These results suggest that inhibiting the roles of GluK1 shows an antiepileptic effect.

### 2.7 AC1 Signal Pathway was Involved in Seizure‐Like Activity

**Figure 7:**
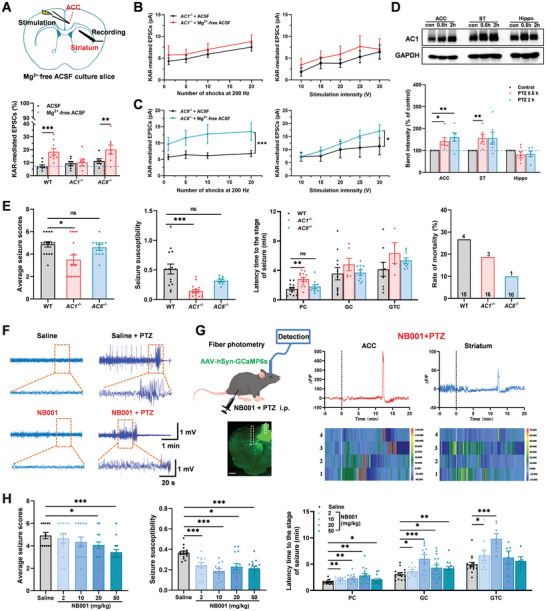
Calcium‐stimulated adenylyl cyclase subtype 1 (AC1) was involved in seizure‐like activity. A) Upper: schematic diagram showing the placement of stimulating electrode in the ACC and recording electrode in the dorsal striatum in Mg^2+^‐ACSF culture slice. Bottom: statistical results showing that KAR‐mediated EPSCs were not increased after perfusing Mg^2+^‐free ACSF for 2 h in *AC1* ^−/−^ mice, but increased in WT and *AC8* ^−/−^ mice (WT: *n* = 9 neurons/3 mice; *AC1* ^−/−^, *n* = 7 neurons/3 mice; *AC8* ^−/−^, *n* = 6–8 neurons/3 mice; **p < 0.01 and ***p < 0.001, unpaired *t*‐test). B) Statistical results showed the amplitude of the KAR‐mediated EPSCs by repetitive stimulations (200 Hz) (*F*_(1, 48)_ = 1.78, *P* > 0.05, two‐way ANOVA) and the input‐output relationship (*F*_(1, 60)_ = 1.81, *p *> 0.05, two‐way ANOVA) after perfusing Mg^2+^‐free ACSF for 2 h in the *AC1* ^−/−^ mice. C) Statistical results show the amplitude of the KAR‐mediated EPSCs by repetitive stimulations (200 Hz) (*F*_(1, 48)_ = 13.53, *p *< 0.001, two‐way ANOVA) and the input‐output relationship (*F*_(1, 55)_ = 4.53, *p *< 0.05, two‐way ANOVA) after perfusing Mg^2+^‐free ACSF for 2 h in the *AC8* ^−/−^ mice. D) Representative and statistics western blots of AC1 expression in total homogenates of the ACC, striatum, and hippocampus, *n* = 5–6. E) The PTZ‐induced seizure severity scores, seizure susceptibilities, the latency time to the stage of seizures, and the rate of mortality in the WT (*n* = 15 mice), *AC1* ^−/−^ (*n* = 16 mice), and *AC8* ^−/−^ (*n* = 10 mice). F) EEG recording showed that NB001 reduced the amplitude and firing rate of EEG compared with the saline group. G) The ΔF/F of representative trace (upper) and heatmaps (bottom) show that Ca^2+^ signals of the ACC (left) and striatum (right) in mice injected with NB001 + PTZ. The colored bar on the right of heatmaps indicates ΔF/F(%), *n* = 4 mice, scale bar: 200 µm. H) The PTZ‐induced seizure severity scores (*F*_(4, 62)_ = 3.018.53, *p *< 0.05, one‐way ANOVA) and seizure susceptibilities (*F*_(4, 64)_ = 8.050, *p *< 0.0001, one‐way ANOVA) were significantly decreased in the i.p. injected AC1 inhibitor NB001 (Saline, *n* = 12 mice; 2 mg/kg, *n* = 12 mice; 10 mg kg^−1^, *n* = 12 mice; 20 mg kg^−1^, *n* = 15 mice; 50 mg kg^−1^, *n* = 16 mice). The latency time to the stage of PC, GC, and GTC seizures induced by PTZ was increased in NB001‐injected mice (*F*_(4, 169)_ = 10.10, *p *< 0.0001, two‐way ANOVA). n.s means no significant difference, *p < 0.05, **p < 0.01, and ***p < 0.001, error bars indicated SEM.

Adenylyl cyclase is a key downstream signal molecular for glutamate receptors in the brain cortex, including adenylyl cyclase subtype 1 (AC1) and subtype 8 (AC8).^[^ ^49^, ^50^ ^]^ We then studied the possible role of adenylyl cyclase in the upregulation functions of KARs after seizure‐like activity. We found that the KAR‐mediated EPSCs were unchanged in *AC1* ^−/−^ mice after perfusing with Mg^2+^‐free ACSF which induced seizure‐like activities compared with the normal ACSF group (**Figure** **7**A). In addition, KAR‐mediated EPSCs did not significantly increase after repetitive stimulation and the input‐output curves did not change in *AC1* ^−/−^ mice after seizure‐like activities (Figure 7B). However, in *AC8* ^−/−^ mice, the KAR‐mediated EPSCs were significantly increased after perfusing with Mg^2+^‐free ACSF (*p* < 0.05, Figure 7A). KAR‐mediated EPSCs also upregulated after repetitive stimulation and the input‐output curves shifted to the left in *AC8* ^−/−^ mice after seizure‐like activities (Figure 7C). These results indicate that AC1, not AC8, is required for the upregulation of KAR‐mediated EPSCs after seizure‐like activities in vitro.

By the western blot experiments, we found the expression of the AC1 was increased in the ACC and striatum over time after the PTZ‐induced in vivo seizure (Figure 7D). Next, we tested whether inhibition of AC1 functions could reduce behavioral seizures in vivo. As shown in Figure 7E, the PTZ‐induced seizure severity scores and susceptibility were significantly decreased in *AC1* ^−/−^ mice, but not in *AC8* ^−/−^ mice. We found that the time to PC stage in the *AC1* ^−/−^ mice was extended compared with the wild‐type mice, but not changed in *AC8* ^−/−^ mice. There was a decrease in the death ratios in *AC1* ^−/−^ mice and *AC8* ^−/−^ mice after the PTZ model (Figure 7E).

Then, we tested the effect of AC1 inhibitor NB001 on PTZ‐induced seizure behaviors in wild‐type mice. We pretreated using either saline and NB001 (2, 10, 20, and 50 mg kg^−1^, i.p.) 30 min before PTZ injection. By EEG recording, we found that the NB001(10 mg kg^−1^, i.p.) reduced the amplitude and firing rate of EEG compared with the saline group (Figure 7F). Combined with fiber‐photometry, we found that the NB001(10 mg kg^−1^, i.p.) reduced the frequency and intensity of Ca^2+^ spikes both in the ACC and the striatum after the PTZ‐induced model. In addition, NB001 also delays the occurrence time of Ca^2+^ spikes, which may related to the different phases of seizure behavior (Figure 7G). NB001 dose‐dependently reduced the severity of seizure behaviors and decreased seizure susceptibility in the PTZ‐induced seizure mice. Especially, NB001 significantly extended the latency time to both PC and GC stages in the PTZ‐induced seizure mice (Figure 7H). Together, these results indicate that the KAR‐AC1 signal pathway is involved in seizure‐like activity, and AC1 inhibitor NB001 has an effective therapeutic effect on seizure behaviors.

## 3 Discussion

The ACC is a key cortical region for emotional responses and sensory perception (see Bliss et al., 2016 for review). However, less is known about the specific contribution of different output projections from the ACC. In the present study, we revealed the characteristics of synaptic transmission and the roles of the ACC to striatum connection. We found that there were direct glutamatergic projections from the different layers of the ACC to the dorsal striatum neurons, which were mainly mediated by AMPA/KA receptors. There is little KA receptor contribution to the basal synaptic transmission in the corticostriatal connection from the ACC. Interestingly, KAR‐mediated EPSCs were increased in the Mg^2+^‐free ACSF‐induced seizure‐like activities in vitro and PTZ‐induced seizure in vivo. Inhibiting the activities of the GluK1 receptor or corticostriatal connection from ACC attenuated seizure behaviors. Calcium‐stimulated AC1 was involved in the role upregulation of KAR in the ACC‐striatum synapses after seizure. Finally, PTZ‐induced seizure activities were attenuated in *AC1^−/−^* mice, and in mice pretreated with AC1 inhibitor NB001. Our results provide direct evidence that the corticostriatal projection from the ACC is involved in seizure modulation and the KAR‐AC1 signal pathway is a potential target for the treatment of epilepsy.

Previous studies have reported that the striatum receives excitatory projections from different cortical areas.^[^ ^38^, ^39^ ^]^ In our recent study, we found that there is a direct projection from the ACC to the dorsal striatum. The projection fibers from the ACC are mainly distributed in the dorsomedial striatum with a few in the dorsolateral striatum. These results are similar to previous reports that the dorsomedial striatum mainly received projection from the medial prefrontal cortex (mPFC), and the dorsolateral striatum mainly received projection from the sensorimotor cortex.^[^ ^38^, ^51^ ^]^ In addition, we found that response latencies to the projections from different layers of ACC are different. These findings suggest that different layers of the ACC neurons form distinct synaptic connections with striatum neurons. Our results also found that the corticostriatal projection from the ACC is glutamatergic. This is consistent with previous anatomic studies that corticostriatal projections are glutamatergic fibers.^[^ ^38^, ^39^ ^]^ KA receptors contribute to fast synaptic transmission in different brain regions, such as the spinal cord, the ACC, the insular cortex, and the amygdala.^[^ ^28^, ^43^, ^52^, ^53^ ^]^ However, to our surprise, there were few KAR‐mediated EPSCs in the ACC‐striatum pathway, even after high‐frequency repetitive stimulation. KARs may be mostly located extrasynaptically or near the soma, since puff‐application of glutamate or ATPA‐induced KAR‐mediated EPSCs in the striatum neurons. These findings also suggest that central glutamatergic synapses are heterogeneous, and there are at least two different types of glutamatergic synapses in the adult brain: one contains pure postsynaptic AMPA receptors, and another one contains both AMPA and KA receptors.^[^ ^54^ ^]^ In addition, there are also several reports of silent synapses in the adult cortex, including possibly ACC.^[^ ^55^, ^56^ ^]^

Previous studies have indicated that KA receptors are involved in seizure processes.^[^ ^9^, ^13^ ^]^ We found that KAR‐mediated synaptic transmission is potentiated after the seizure‐like activities induced by the Mg^2+^‐free ACSF in vitro and injection of pentylenetetrazole in vivo. Previous studies in the hippocampus have shown that the expression of KA receptors was increased in the hippocampal astrocytes after kainate acid‐induced seizure activity.^[^ ^57^ ^]^ We also find the the expression of KA receptors is increased in the ACC and striatum after pTZ‐induced seizure. Therefore, seizure‐like activities may increase the expression of KA receptors in the corticostriatal projection from the ACC. In addition, KA receptors are known to exert modulatory roles in the release of excitatory or inhibitory neurotransmitters.^[^ ^28^, ^42^, ^53^ ^]^ Therefore, it is likely that KA receptors might contribute to the imbalance between excitatory and inhibitory synaptic transmissions in the seizure. In the present study, we found that inhibiting the ACC‐striatum circuit reduced PTZ‐induced seizure activities. These results suggest KA receptor mediated the synaptic transmissions in the corticostriatal connection involved in the seizure activities.

GluK1 has been suggested as one of the possible drug targets for treating epilepsy.^[^ ^18^ ^]^ In this work, we found that seizure activities induced by PTZ were attenuated in *GluK1^−/−^* mice. Injection of GluK1 antagonist UBP310 both in the ACC and striatum can decrease seizure behaviors. This result supports the idea that GluK1 is a potential anti‐epileptic drug target. LY37770 and LY382884, 2 selective GluK1 antagonists, blocked hippocampal epileptiform activities induced by pilocarpine or electrical stimulation in rats, both in vitro and in vivo.^[^ ^18^ ^]^ In addition, topiramate, a clinically used medicine, is thought to produce an anti‐epileptic effect by inhibiting GluK1 function.^[^ ^58^, ^59^ ^]^

Calcium‐stimulated adenylyl cyclase subtype 1 (AC1) plays a crucial role in downstream glutamate receptors, including the KA receptor.^[^ ^29^, ^60^ ^]^ Compared with *GluK1^−/−^* mice, *AC1^−/−^* mice showed more effectively attenuated seizure activities. Moreover, like *AC1^−/−^* mice, intraperitoneal injection of NB001 reduced behavioral seizures. NB001 can also significantly delay the latency time to stages in the PTZ‐induced seizure mice. These results proved a possibility for NB001 as an anti‐epileptic medicine. As compared with the current anti‐epileptic drugs, AC1 targeted selective inhibitor NB001 is very safe in both animals and humans.^[^ ^61^ ^]^ NB001 might be another successful choice for antiepileptic therapy in the future.

## 4 Experimental Section

### Animals

Adult C57BL/6 male mice were purchased from the Experimental Animal Center of Xi'an Jiaotong University (8‐12 weeks old). CaMKIIα‐Cre mice were purchased from Jaxlab (JAX:005359). *GluK1^−/−^* mice were obtained as gifts from Dr. Stephen F. Heinemann (Salk Institute, San Diego, CA) and were maintained on a C57BL/6 background. *AC1^−/‐^* mice and *AC8^−/‐^* mice were a gift from Dr. Daniel R. Storm (University of Washington, Seattle, WA) and were maintained on a C57BL/6 background. All mice were maintained on a 12‐h light/dark cycle with food and water provided ad libitum. All the mice were male in the whole experiment. The Animal Care and Use Committee of Xi'an Jiaotong University (IDs: No. 2020–576) approved all experiment protocols.

### VISoR Imaging

For tracing ACC inputs and outputs projections, 200 nL of AAV2/9‐hSyn‐EGFP‐2a‐TVA‐2a‐RVG‐WPREs‐pA (2.0 × 10^12^ genomics copies per mL, Brainvta, Wuhan, China) as the helper virus was injected into the right ACC (anteroposterior +1.0 mm, mediolateral ‐0.4 mm, and dorsoventral ‐1.5 mm). Three weeks after AAV viral expression, 200 nL of RV‐EnvA‐ΔG‐DsRed (2.0 × 10^8^ genomic copies per mL, Brainvta, Wuhan, China) was injected into the same site of ACC. For ACC‐striatum sparse labeling, 50 nL of AAV2/9‐CMV‐Cre diluted 1000 times (2.0 × 10^12^ genomics copies per mL, Brainvta, Wuhan, China) and AAV2/9‐EF1α‐DIO‐EYFP‐EYFP (2.0 × 10^12^ genomics copies per mL, Brainvta, Wuhan, China) were separately injected into the unilateral ACC.

Viral injection procedures were performed as previously described.^[^ ^62^ ^]^ Briefly, the virus was injected into the ACC location with equal speed by micro‐syringe pump (23 nL min^−1^; Nanoject II, DRUMMOND). Mice were allowed to survive for ≈3 weeks for AAV virus expression and 7 days for RV virus expression. The mice were then deeply anesthetized with 2% isoflurane and perfused intracardially with 0.01 m phosphate‐buffered saline (PBS, pH 7.4) followed by 4% w/v paraformaldehyde (PFA, pH 7.4). To allow penetration of fixation solution, the brain was removed and incubated in 4% hydrogel monomer solution (HMS, 4% w/v acrylamide, 0.05% w/v bisacrylamide, 1× PBS, 4% w/v PFA, 0.25% VA‐044 thermal initiator, and distilled water) at 4 °C for 2 days. The whole brain of the mouse was embedded with an equal volume mixture of 4% HMS and 20% bovine serum albumin (BSA) at 37 °C for 4 h. The embedded sample was cut into 300 µm thickness coronal slices by the vibroslicer (Compresstome VF‐300, Precision Instruments). Cleared brain slices obtained by the two‐step PuClear clearing method were imaged at 1 × 1 × 2.5 µm^3^ voxel resolution using the VISoR2 technique as described previously.^[^ ^63^ ^]^ Synchronized beam‐scan illumination and camera‐frame readout generated a stack of image frames of the 45 ° oblique optical sections of the sample with the sample stage moving linearly in the X‐direction. Image volumes of each mouse brain slices were stitched with the custom software to automatically reconstruct the whole brain as described previously.^[^ ^63^ ^]^

### Optogenetics and Chemical Genetics

Anterograde tracer AAV2/9‐hSyn‐hChR2‐EYFP or AAV2/9‐hSyn‐eNpHR‐mCherry was purchased from BrainVTA (Wuhan) Co., Ltd. The virus was injected into 2 sides of the ACC in C57BL/6 adult mice. For in vitro whole‐cell patch‐clamp recording, three weeks after injection of AAV2/9‐hSyn‐hChR2‐EYFP, neurons were illuminated with the same system used for the in vitro whole‐cell patch‐clamp recording (see below). The optical fiber was localized in the recording chamber at 3 mm from the recorded neurons. The ACC neurons were recorded in the current clamp at a holding current allowing maintaining the resting membrane potential. The striatum neurons were fixed at –60 mV in voltage‐clamp mode. The blue light was generated by a 473 nm laser (5 ms) and driven at single, 5 Hz (1 s), 10 Hz (1 s), or 20 Hz (1 s) to activate neurons. An intensity of 15 mW mm^−2^ at the fiber tip was used in all experiments.^[^ ^64^, ^65^ ^]^

For behavioral tests combined with the optogenetics technique, the optical fiber (length, 3 mm; 200 µm core; NA = 0.37; THINKERTECH, Nanjing) was implanted into the striatum (anteroposterior +1.0 mm, mediolateral −1.2 mm, and dorsoventral −2.6 mm from the bregma) two weeks after the virus expression. Two weeks after the mice recovered, the optical fiber was connected to fiber‐coupled lasers to control the frequency and intensity of the light. Mice received blue (473 nm, 2–5 mW, 20 Hz, 5 ms pulse) or yellow light (593 nm, 5–8 mW, constant) illumination during testing. After the experiments, all mice were sacrificed and the whole brain was sectioned to examine the location of virus expression and optical fiber implantation. If virus expression or fiber implantation deviated from the target area, the data were excluded.

For the chemical genetics experiment, Anterograde tracer AAV2/9‐hSyn‐hM3Dq‐EYFP or AAV2/9‐hSyn‐hM4Di‐mCherry (BrainVTA, Wuhan) was injected into 2 side of the ACC in C57BL/6 adult mice. Three weeks after viral expression, CNO (1 mg/kg) was intraperitoneally (i.p.) injected into mice before testing.

### Brain Slice Preparation

Coronal brain slices (300 µm) containing the ACC and the striatum were prepared using our previous methods.^[^ ^65^, ^66^ ^]^ Briefly, mice were anesthetized with isoflurane and sacrificed by decapitation. The whole brain was quickly removed from the skull and submerged in ice‐cold oxygenated (95% O_2_ and 5% CO_2_) artificial cerebrospinal fluid (ACSF) containing (in mM) 124 NaCl, 2.5 KCl, 2 CaCl_2_, 2 MgSO_4_, 25 NaHCO_3_, 1 NaH_2_PO_4_ and 10 glucose). For making coronal brain slices, the brain was glued to the cutting staged tissue slicer (Leica, VT1200S). Slices were transferred to a submerged recovery chamber with oxygenated (95% O_2_ and 5% CO_2_) ACSF at room temperature for at least 1 h.

### In Vitro Whole‐Cell Patch‐Clamp Recording

Experiments were performed in a recording chamber placed in an Olympus BX51W1 microscope with infrared DIC optics for visualization of whole‐cell patch‐clamp recording. Excitatory postsynaptic currents (EPSCs) were recorded from neurons with an Axon 200B amplifier (Axon Instruments) in the striatum and a bipolar tungsten‐stimulating electrode placed in layer V/VI of the ACC delivered stimulation. The recording pipettes (3–5 MΩ) were filled with a solution containing (in mM) 145 K‐gluconate, 5 NaCl, 1 MgCl_2_, 0.2 EGTA, 10 HEPES, 2 Mg‐ATP, and 0.1 Na_3_‐GTP (adjusted to pH 7.2 with KOH, 290 mOsmol). EPSCs were recorded in the voltage‐clamp configuration and the membrane potential was held at −60 mV throughout the experiment. AMPA/KA receptor‐mediated EPSCs were induced by repetitive stimulations with intervals of 30 s in the presence of AP‐5 (50 µM). Both AP‐5 and GYKI 53655 (100 µM) were applied for KA currents. For frequency facilitation, repetitive stimulation was delivered at 200 Hz (5, 10, or 20 shocks). Picrotoxin (100 µM) was always present to block GABA_A_ receptor‐mediated inhibitory synaptic currents in all experiments. Access resistance was 15–30 MΩ and was monitored throughout the experiment. Data were discarded if access resistance changed by 15% during the experiment. Data were filtered at 1 kHz and digitized at 10 kHz using the Digidata 1440A.

The morphological properties of the dorsal striatum neuron were labeled by 0.5% biocytin into the recording solution for patched neurons.^[^ ^43^ ^]^ Briefly, the slices containing biocytin‐labeled cells were fixed with 4% paraformaldehyde in 0.1 m PB and were dehydrated with 30% sucrose. Slices were immunostained with FITC conjugated avidin (1: 200, Jackson) for 2 h at room temperature. The immunofluorescence‐labeled neurons were imaged with a confocal microscope (Fluoview FV1000, Olympus, Tokyo, Japan) using the appropriate filter for FITC.

### Behavioral Seizures Induction: Induction of Seizure‐Like Activity In Vitro

Seizure‐like activity was induced by incubation of slices in Mg^2+^‐free solution as follows (in mM): 124 NaCl, 2.5 KCl, 2 CaCl_2_, 25 NaHCO_3_, 1 NaH_2_PO_4_, and 10 D‐glucose, equilibrated with 95% O_2_ and 5% CO_2_ at the room temperature.^[^ ^45^, ^46^ ^]^ After 10–15 min incubation, the seizure‐like activities were elicited in the neuron in the current‐clamp configuration.

### Behavioral Seizures Induction: Induction of Seizure Activity In Vivo

We performed intraperitoneal administration of 50 mg kg^−1^ pentylenetetrazole (PTZ, Sigma, MO, USA) to induce seizures in vivo as previously reported. Mice were taken from their home cages and placed individually into test cages within corncob‐lined plastic new cages for 30 min before PTZ injection. After injection, mice were immediately returned to the test cage for seizure activity with video recording. Seizure activity, which was usually induced within 15 min of PTZ injection, was observed for 30 min after injection. Seizure behavior was scored based on Racine's scale^[^ ^48^, ^67^ ^]^ stage 0, no seizure; stage 1, hypoactivity, head nodding; stage 2, partial clonus (PC) involving clonic seizure activity affecting face, head, or forelimb; stage 3, generalized clonus (GC), whole body clonus including all four limbs and tail, rearing, or falling; stage 4, generalized tonic‐clonic seizure (GTC), jumping, shrieking, falling over; stage 5, violent convulsions, tonic‐clonic maximal seizures were associated with mice recovered spontaneously; stage 6, tonic seizure followed by death.

Seizure susceptibility was calculated from the latencies to partial clonus (PC), generalized clonus (GC), and generalized tonic‐clonic (GTC) seizures, as previously described.^[^ ^48^, ^67^ ^]^

Seizure susceptibility = 0.2* (1/ latency to PC) + 0.3* (1/latency to GC) + 0.5* (1/ latency to GTC).

### Ca^2+^ Signal Recording in Freely Behaving Mice

For Ca^2+^ recording, 200 nL of anterograde tracer virus AAV2/9‐hSyn‐GCaMP6s‐WPRE‐pA was injected into the unilateral ACC. The optical fibers (length, 3 mm; 200 µm core; NA = 0.37; THINKERTECH, Nanjing) were implanted into the ipsilateral ACC (anteroposterior +1.0 mm, mediolateral −0.3 mm, and dorsoventral −1.4 mm) and striatum (anteroposterior +1.0 mm, mediolateral −1.6 mm, and dorsoventral −2.6 mm). The Fluorescence of GCaMP6s was excited by fiber‐coupled lasers (200 mW, THINKERTECH, Nanjing) delivering a 470 nm light to the ACC or striatum. After the optical fibers were connected, the baseline was recorded for 5 min, and then the PTZ was intraperitoneally injected into mice. The behavior videos and neuronal Ca^2+^ signals of the ACC and striatum were recorded during baseline and 30 min after PTZ injection. NB001 (10 mg kg^−1^) or Saline was injected before 30 min of PTZ. Matlab Mat software was used for data analysis. We derived the values of Ca^2+^ signal changes (ΔF/F) by calculating (F−F0)/F0.

### Western Blot

The brain region that needed to be identified was homogenized in lysis buffer, including 10 mM Tris (pH 7.4), 2 mM EDTA, and 1% SDS, 1× protease inhibitor cocktail. After centrifugation at 14 000 rpm for 20 min at 4 °C, the supernatant was used for total protein analysis and western blotting. Sample protein concentrations were quantified using BCA assay (Beyotime), and electrophoresis of equal amounts of protein and volume (30 µg/20 ul/lane) was performed on 7.5% SDS‐polyacrylamide gel. The quantified protein from tissue lysates was separated by SDS‐PAGE electrophoresis and then transferred to polyvinylidene difluoride membranes. After blocking with 5% skim milk for 1 h at room temperature, blots were probed with anti‐GluA1 (1:1000; Millipore, US), anti‐GluA1‐S831 (1:500; Millipore, US), anti‐GluA1‐S845 (1:500; Millipore, US), anti‐GluN2A (1:2000; Millipore, US), anti‐GluN2B (1:1000; Millipore, US), anti‐GluK1 (1:1000; Millipore, US), anti‐GluK2/3 (1:2000; Millipore, US), anti‐GluK4 (1:500; Millipore, US), anti‐ GluK5 (1:500; Millipore, US), or anti‐AC1 (1:500; Millipore, US) polyclonal antibodies overnight at 4 °C. To verify equal loading, membranes were also probed with anti‐GAPDH antibody as an internal reference (1:4000; Sigma, US). The membranes were incubated with a horseradish peroxidase‐conjugated goat anti‐rabbit or anti‐mouse IgG (1:5000; Millipore, US) for 1 h at room temperature, and the bands were visualized by an ECL system (GE Healthcare, US). Use appropriate exposure time to ensure that the signals are in the linear range. Signals were quantified using ImageJ software. Membranes were used again for another similar molecular weight primary antibody by stripping buffer (62.5 mM Tris‐HCl (pH 6.8), 2% SDS, 100 mM β‐mercaptoethanol) followed blocking. Signals were quantified using ImageJ software.

### EEG Recording

Briefly, mice were anesthetized by inhalation of isoflurane (1%–3%). The head was fixed into a stereotaxic frame, and an incision was made over the skull to expose the surface. An EEG electrode (Jiangsu Brain Medical Technology Co. Ltd, China) with 2 channels was implanted into the subiculum for EEG recording. Two screws were placed in the skull over the cerebellum to serve as the reference and ground electrodes and to secure the dental cement, together with a third screw over the sensorimotor cortex. Dental adhesive resin cement (Super‐bond C&B, Japan) was used to stick the metal head bar to the exposed skull, to ensure the security of the head‐fixed position when the head bar was held firmly by the behavioral apparatus during recording. The mice were given at least 1 week to recover after the implantation of the EEG electrodes. EEG recordings were performed with a GeoStudio System (Jiangsu Brain Medical Technology Co. Ltd, China). EEG data were recorded from the implanted microwire electrode arrays. The analog signals were filtered by a band‐pass filter set between 0.3 and 250 Hz. The local field potentials were recorded at a sampling rate of 1 kHz s^−1^. EEG data were analyzed by MATLAB. The onsets of seizures were confirmed by both EEG signal and video recordings according to Racine's classification and seizure discharges. For EEG recording, epileptiform discharges were recognized by their large amplitudes (≥3 times of background signals), and repetitive single,or complex spike waveforms and were confirmed using video recordings.

### Immunochemistry

Injection of the AAV2/R‐hSyn‐mCherry virus in the striatum labeled the ACC neurons. After three weeks, mice were deeply anesthetized and perfused with ice‐cold phosphate‐buffered saline (0.1 M PBS) containing 4% paraformaldehyde. Next, the brain was removed and immersed in 4% paraformaldehyde at 4 °C for 24 h. After dehydration in 30% (w/v) sucrose overnight at 4 °C, a series of 30 µm coronal slices across the ACC and striatum were cut using a freezing microtome (Leica CM1950, Germany). After three washes with PBS for 10 min each, the slices were permeabilized with 0.3% Triton X‐100 in PBS containing 2% BSA for 5 min at room temperature. After 2 h of blocking with 10% FBS in PBS, the slices were incubated with primary antibodies at 4 °C overnight. The following primary antibodies were used as indicated: anti‐CaMKII (1:200, ab134041, Abcam) and anti‐GAD67 (1:200, 33379, Signalway Antibody). After three washes with blocking solution, the slices were incubated with secondary antibodies (Alexa Fluor 488 donkey anti‐rabbit IgG (H + L), A‐21206 for 1 h. After three washes, the slices were mounted on slides with DAPI/Antifade solution (S7113, Sigma). All antibodies were diluted in PBS containing 5% (v/v) normal donkey serum (NDS), 0.3% (v/v) Triton X‐100, 0.05% (w/v) NaN3 and 0.25% (w/v) carrageenan (PBS‐NDS, pH 7.4) overnight at 4 °C. Finally, all sections were mounted on glass slides and observed using a laser scanning confocal microscope (FV1000, Olympus, Japan) or a slide scanner (Slideview VS200, Olympus) with the appropriate filters. Images were analyzed with ImageJ (National Institutes of Health).

### Chemicals and Drug Application

D (‐)‐2‐amino‐5‐phosphonopentanoic acid (AP‐5), 6‐Cyano‐7‐nitroquinoxaline‐2,3‐doine (CNQX), 1‐(4‐Aminophenyl)‐3‐methylcarbamyl‐4‐methyl‐3,4‐dihydro‐7,8‐methylenedioxy‐5H‐2,3‐benzodiazepine hydrochloride (GYKI 53665), (RS)‐2‐amino‐3‐(3‐hydroxy‐5‐tert‐butylisoxazol‐4‐yl) propanoic acid (ATPA), 4‐AP, TTX, and UBP310 were obtained from Tocris Cookson (Bristol, UK). Picrotoxin (PTX) and pentylenetetrazole (PTZ) were obtained from Sigma Aldrich (Canada). NB001 was provided by NeoBrain Pharmac Inc (Canada). Drugs were prepared as stock solutions for frozen aliquots at −20  °C. All these drugs were diluted from the stock solution to the final desired concentration in the ACSF before being applied to the perfusion solution. In some experiments, a picopump (WPI pneumatic picopump, Sarasota, FL) was used for puff application of glutamate and ATPA. Before establishing whole‐cell recording, the drug application pipette was moved beside the neuron using a micromanipulator (Sutter MP‐285, Novato, CA). The tip of the pipette was 50 µm away from the recorded neuron. The diameter of the drug application pipette tip was 3–4 µm. The pressure and duration of the puff were 15 psi and 100 ms, respectively.^[^ ^44^, ^68^ ^]^ For experiments requiring drug administration to the brain region, the drug cannula was implanted at the ACC and striatum in mice one week in advance.

### Statistical Analysis

All data were presented as the means ± standard error of the mean (SEM). The numbers of mice, slices, or cells analyzed were indicated in the figure legends. Electrophysiology Data were collected and analyzed with Clampex 10.2 and Clampfit 10.2 software (Molecular Devices). Statistical comparisons were made with the two‐tailed unpaired or paired Student's *t*‐test for comparison between the two groups. For comparison among the three groups, we used one‐way analysis of variance ANOVA or two‐way ANOVA (Student‐Newmann‐Keuls or Tukey test was used for post‐hoc comparisons). All statistical analyses were carried out with GraphPad Prism 8.0 (GraphPad Software), In all cases, *p *< 0.05 was considered statistically significant.

## Conflict of Interest

The authors declare that they have no competing interests.

## Author Contributions

X.H.L. and M.Z. conceived the project and designed the experiments. X.H.L., T.M., Q.Y.C., and S.H. performed the electrophysiological experiments. X.H.L., W.S., Z.X.Z., Z.Z., and M.X. performed the behaviors, virus injection, and optogenetic experiments. J.S.L. and J.M.C. performed the molecular experiments. W.S., M.X., F.X., and G.Q.B. performed the immunohistochemical and morphological experiments. X.H.L., M.Z. W.S., Q.Y.C., B.‐K.K., and G.L.C. drafted the manuscript and finished the final version of the manuscript. All authors have read and approved the final manuscript.

## Supporting information

Supporting Information

## Data Availability

The data that support the findings of this study are available from the corresponding author upon reasonable request.
