# Supplementary material for: Increased GluK1 Subunit Receptors in Corticostriatal Projection from the Anterior Cingulate Cortex Contributed to Seizure‐Like Activities
Source: Adv Sci (Weinh). 2024 Sep 3;11(39):2308444. doi: 10.1002/advs.202308444 (PMC11497107; doi:10.1002/advs.202308444)
Supplement: Supplementary file 1 — Supporting Information [file ADVS-11-2308444-s001.pdf]

ADVANCED SCIENCE
Open Access

Supporting Information

for *Adv. Sci.*, DOI 10.1002/advs.202308444

Increased GluK1 Subunit Receptors in Corticostriatal Projection from the Anterior Cingulate Cortex Contributed to Seizure-Like Activities

*Xu-Hui Li, Wantong Shi, Zhi-Xia Zhao, Takanori Matsuura, Jing-Shan Lu, Jingmin Che, Qi-Yu Chen, Zhaoxiang Zhou, Man Xue, Shun Hao, Fang Xu, Guo-Qiang Bi, Bong-Kiun Kaang, Graham L. Collingridge and Min Zhuo**

The data of Western blot detection the receptors in PTZ model mice

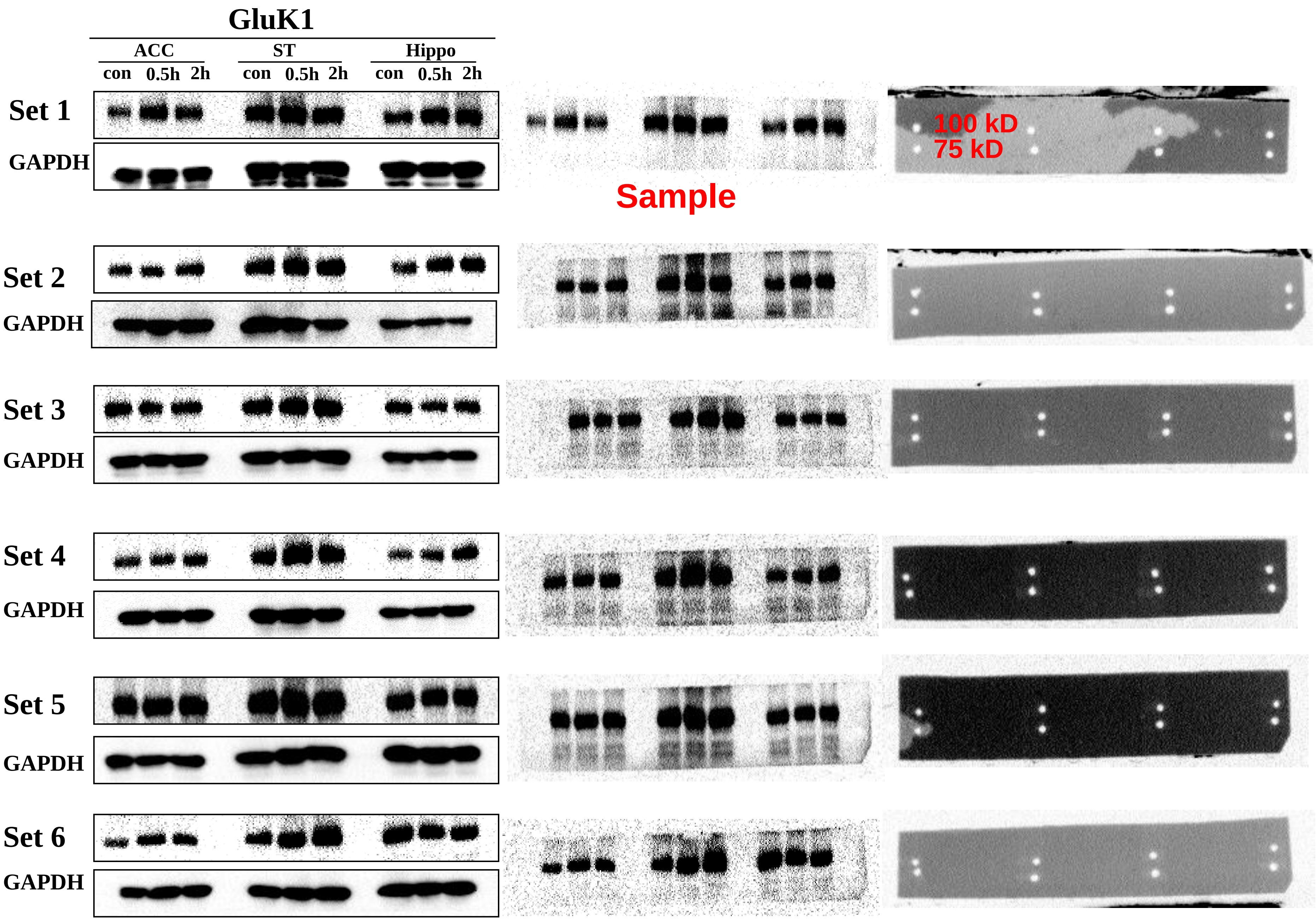
GluK1
ACC
ST
Hippo
con
0.5h
2h
con
0.5h
2h
con
0.5h
2h
Set 1
GAPDH
Set 2
GAPDH
Set 3
GAPDH
Set 4
GAPDH
Set 5
GAPDH
Set 6
GAPDH
Sample
100 kD
75 kD

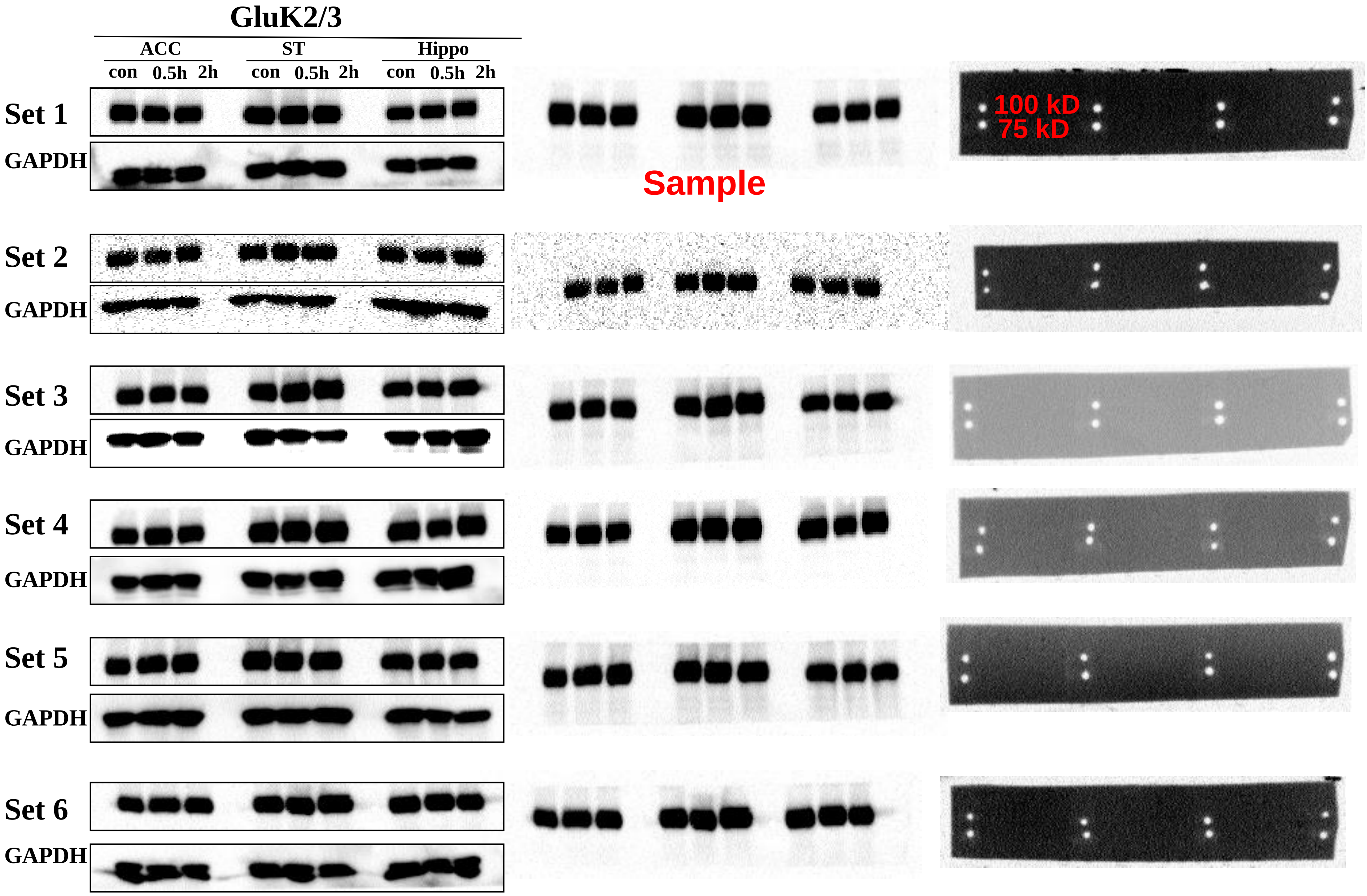
GluK2/3
ACC
ST
Hippo
con 0.5h 2h
con 0.5h 2h
con 0.5h 2h
Set 1
GAPDH
Set 2
GAPDH
Set 3
GAPDH
Set 4
GAPDH
Set 5
GAPDH
Set 6
GAPDH
Sample
100 kD
75 kD

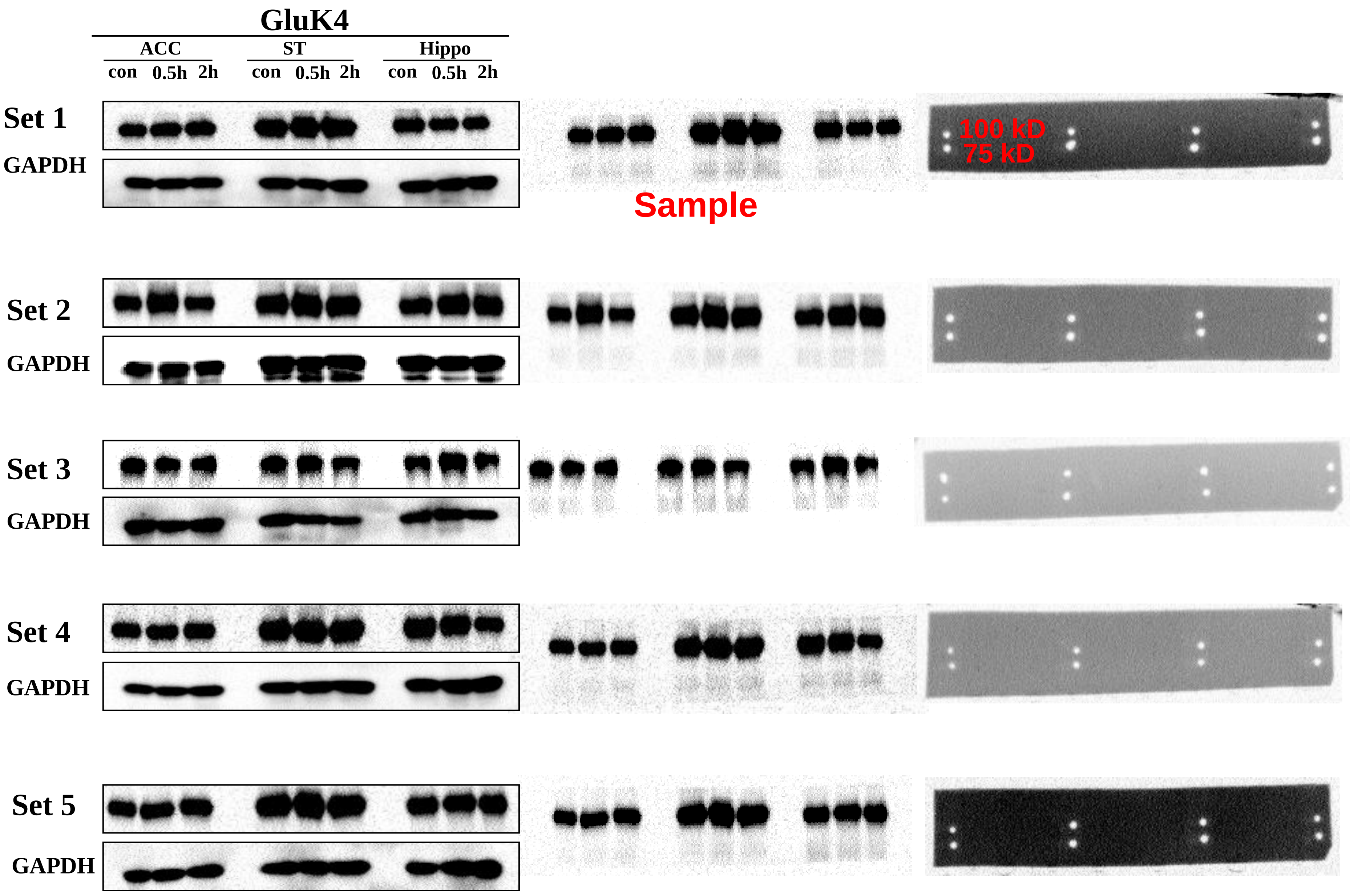
GluK4
ACC
ST
Hippo
con 0.5h 2h
con 0.5h 2h
con 0.5h 2h
Set 1
GAPDH
Set 2
GAPDH
Set 3
GAPDH
Set 4
GAPDH
Set 5
GAPDH
100 kD
75 kD
Sample

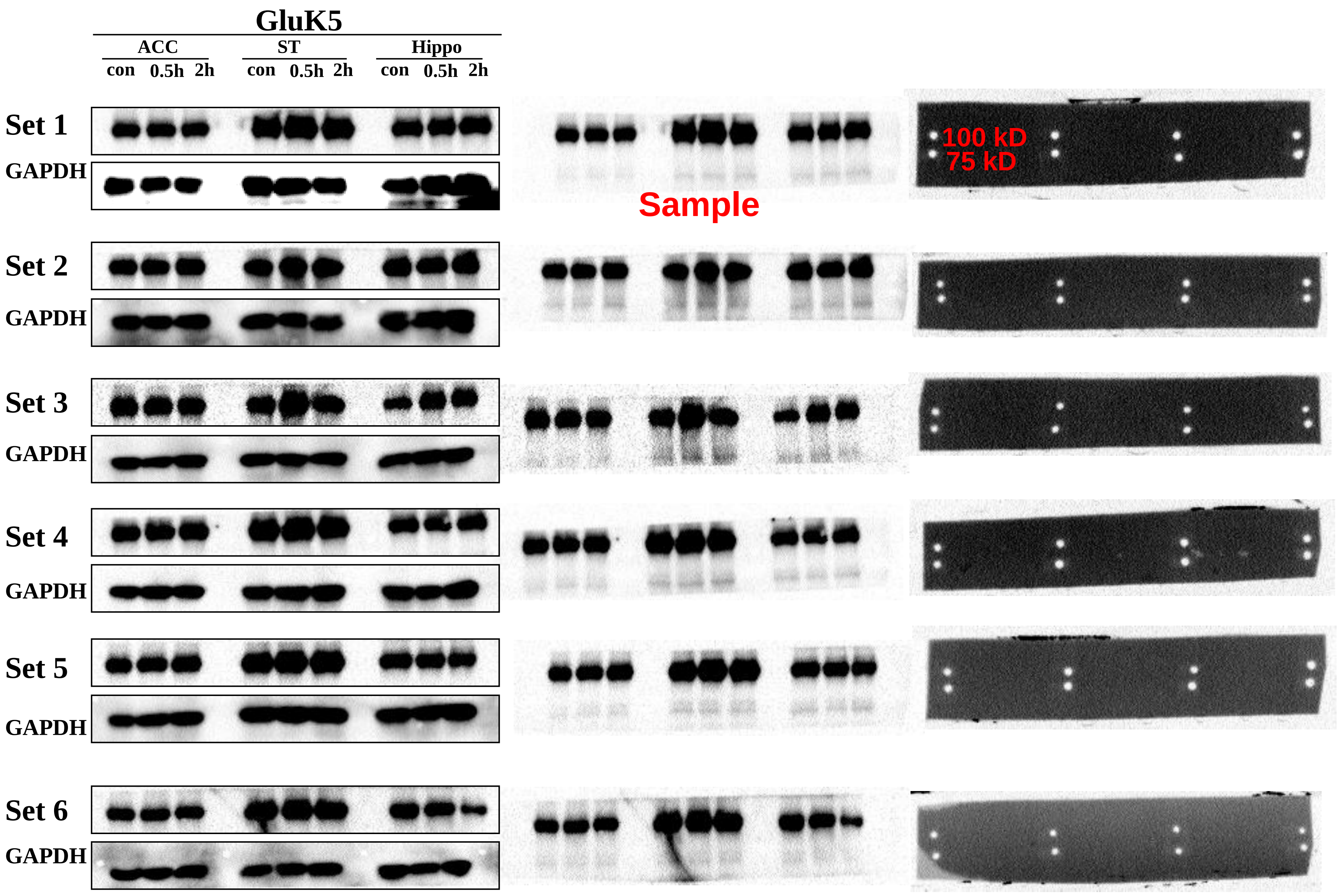
GluK5
ACC
ST
Hippo
con 0.5h 2h
con 0.5h 2h
con 0.5h 2h
Set 1
GAPDH
100 kD
75 kD
Sample
Set 2
GAPDH
Set 3
GAPDH
Set 4
GAPDH
Set 5
GAPDH
Set 6
GAPDH

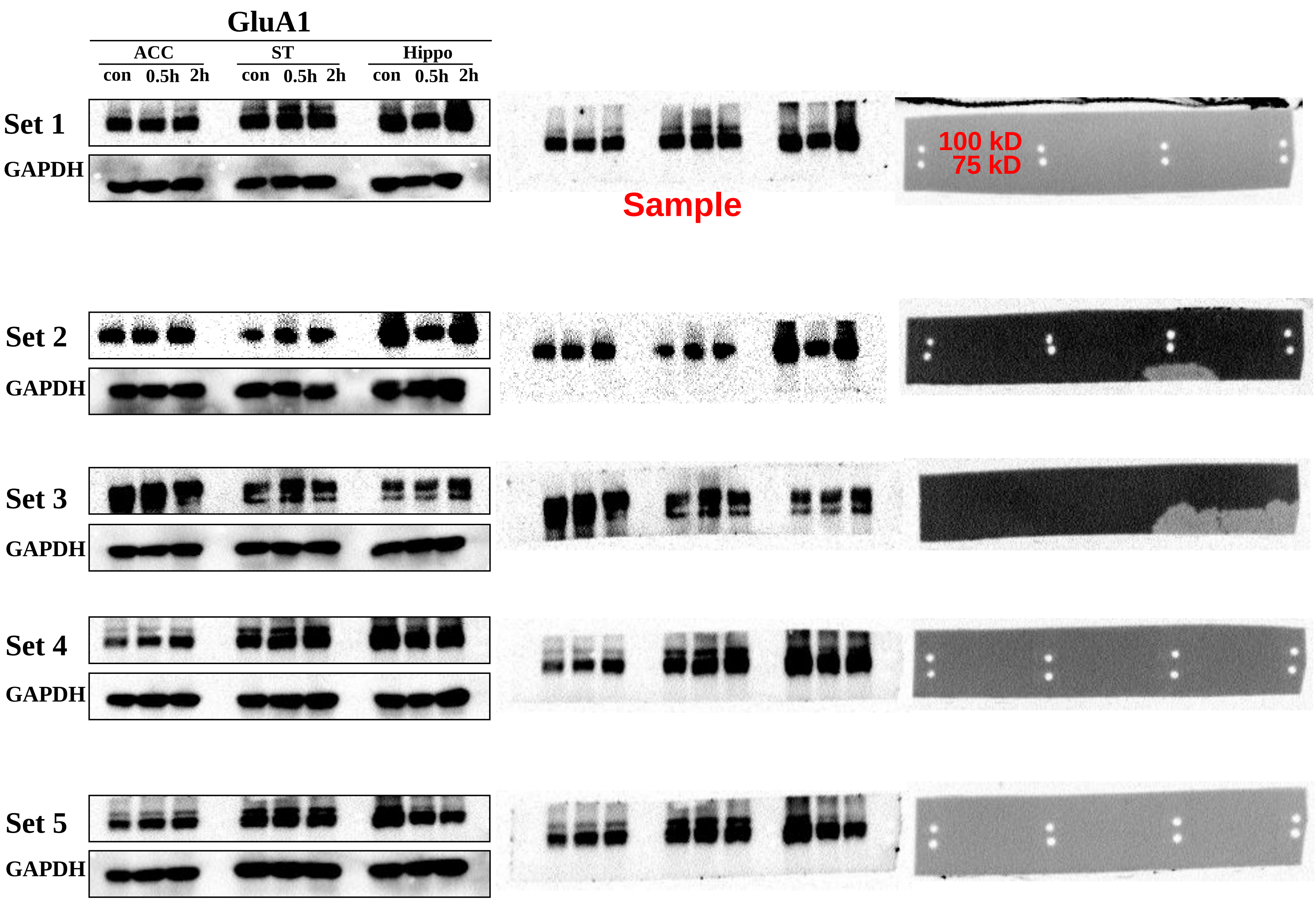
GluA1
ACC
ST
Hippo
con 0.5h 2h
con 0.5h 2h
con 0.5h 2h
Set 1
GAPDH
100 kD
75 kD
Sample
Set 2
GAPDH
Set 3
GAPDH
Set 4
GAPDH
Set 5
GAPDH

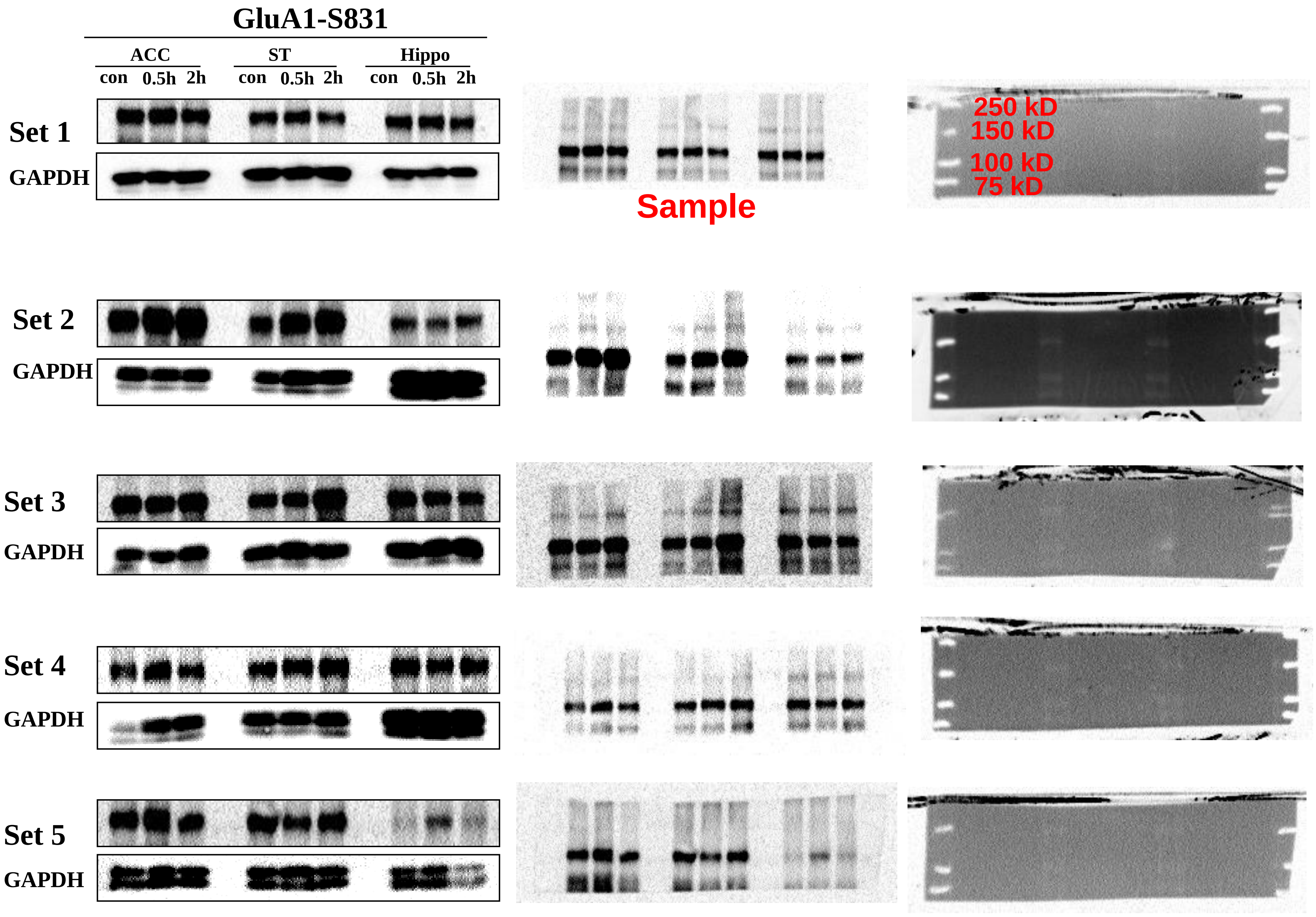
GluA1-S831
ACC
ST
Hippo
con
0.5h
2h
Set 1
GAPDH
Set 2
Set 3
Set 4
Set 5
Sample
250 kD
150 kD
100 kD
75 kD

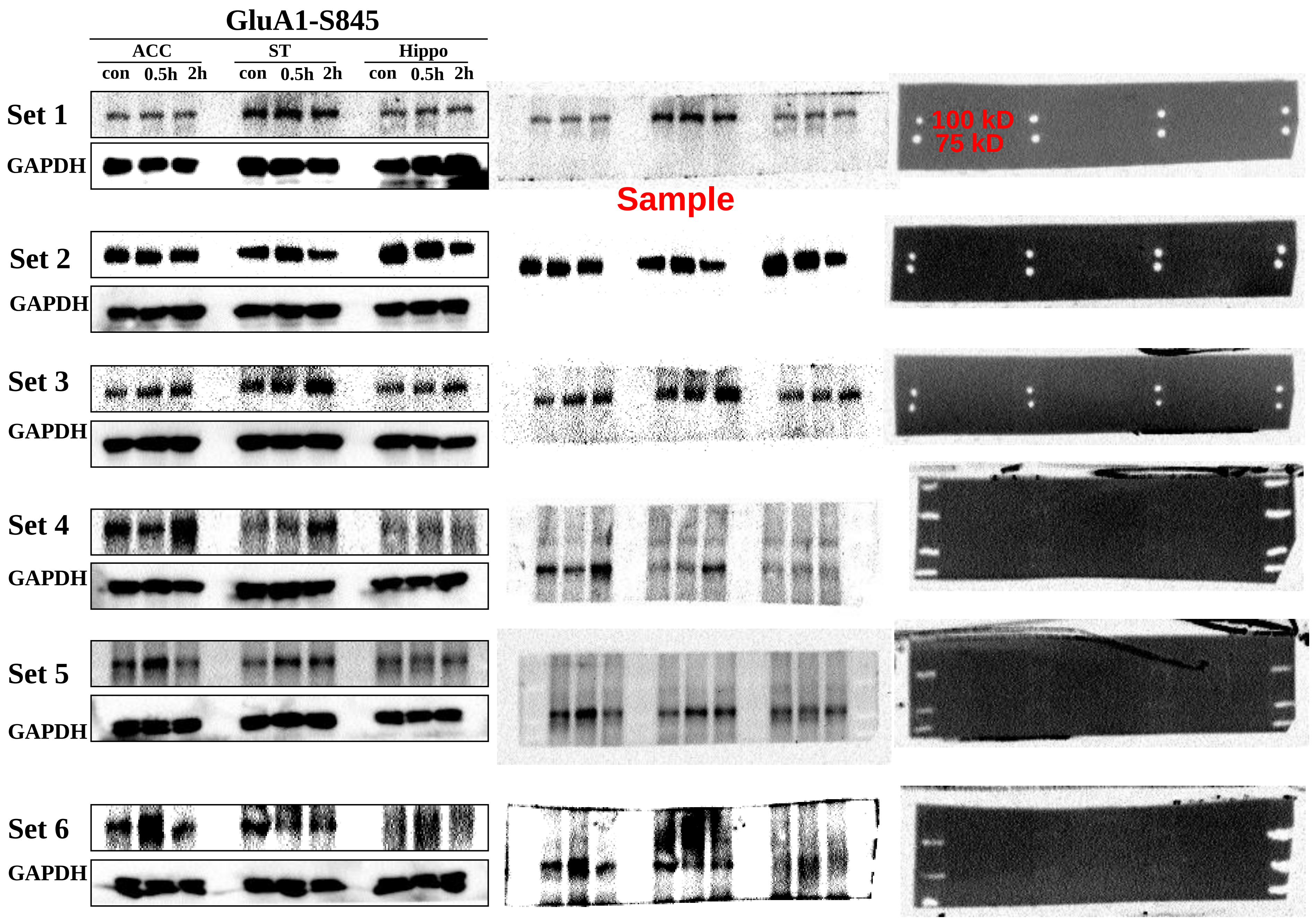
GluA1-S845
ACC
ST
Hippo
con
0.5h
2h
Set 1
GAPDH
Set 2
Set 3
Set 4
Set 5
Set 6
Sample
100 kD
75 kD

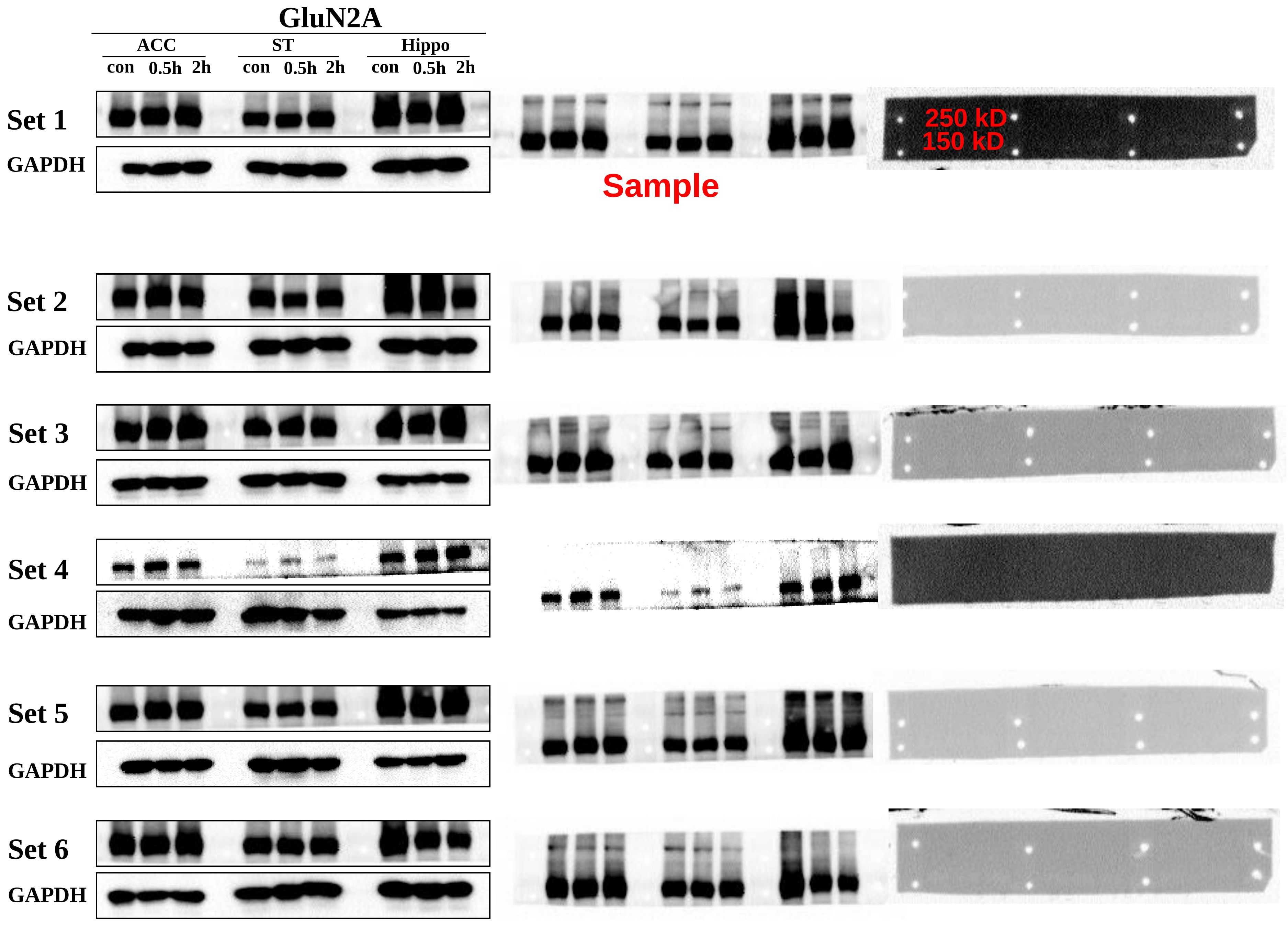
GluN2A
ACC
ST
Hippo
con
0.5h
2h
Set 1
GAPDH
250 kD
150 kD
Sample
Set 2
Set 3
Set 4
Set 5
Set 6

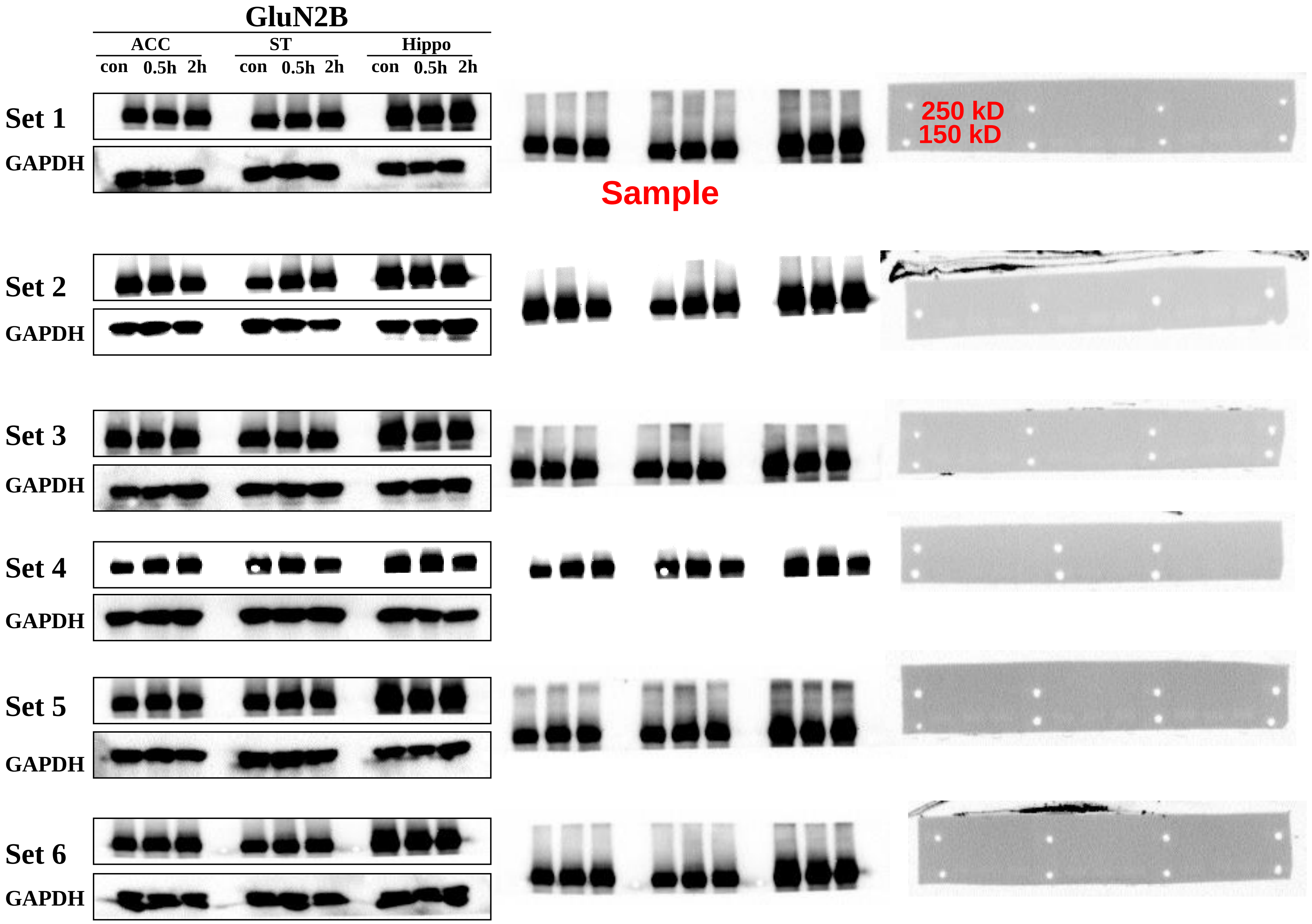
GluN2B
ACC
ST
Hippo
con 0.5h 2h
con 0.5h 2h
con 0.5h 2h
Set 1
GAPDH
Set 2
GAPDH
Set 3
GAPDH
Set 4
GAPDH
Set 5
GAPDH
Set 6
GAPDH
250 kD
150 kD
Sample

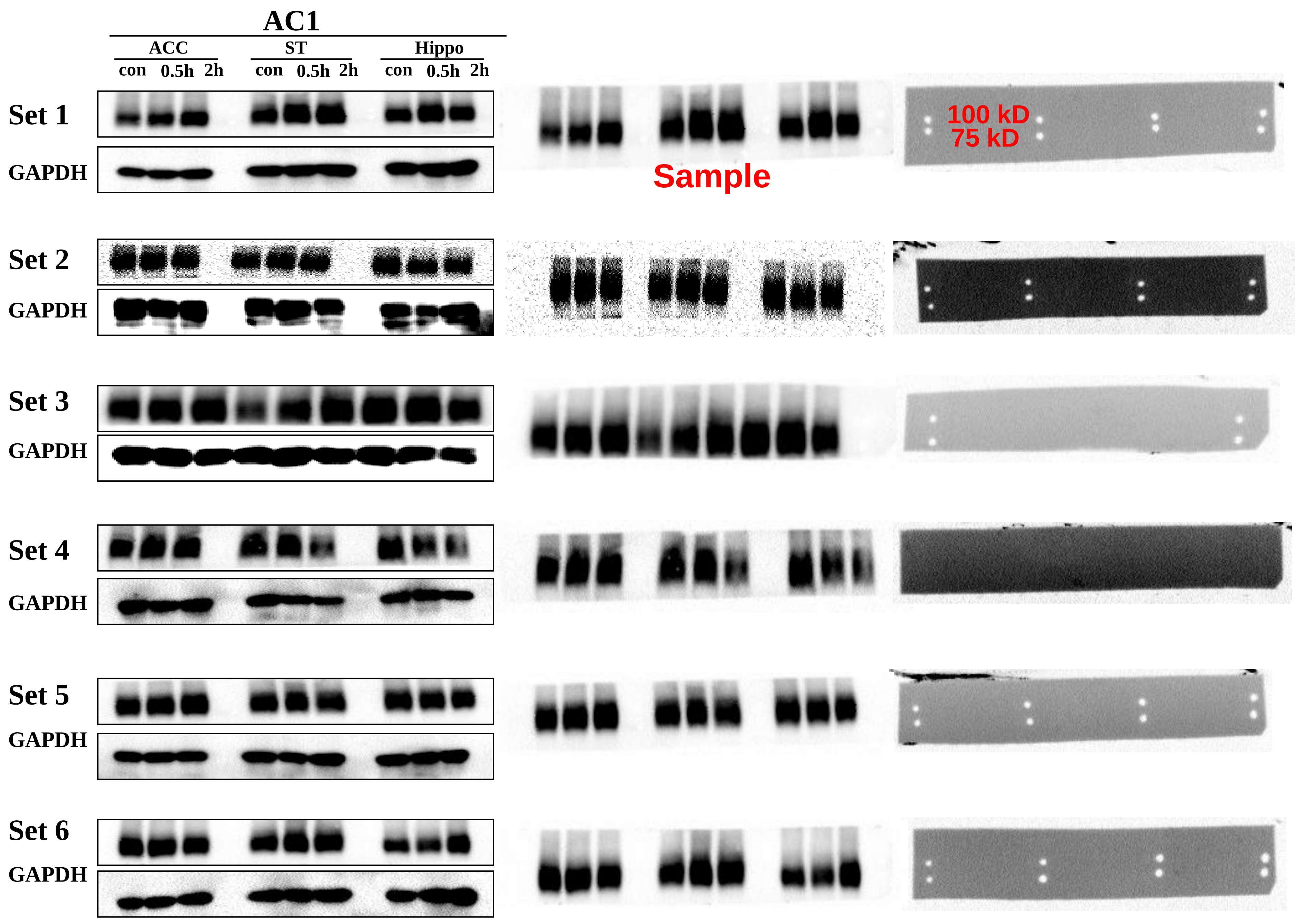
AC1
ACC
ST
Hippo
con
0.5h
2h
con
0.5h
2h
con
0.5h
2h
Set 1
GAPDH
Set 2
GAPDH
Set 3
GAPDH
Set 4
GAPDH
Set 5
GAPDH
Set 6
GAPDH
Sample
100 kD
75 kD
